# Zwitterion‐Stabilized Superlattices and Frank‐Kasper Phases in Charged Block Copolymers for Mechanically Robust Dielectric Soft Materials

**DOI:** 10.1002/advs.202507115

**Published:** 2025-06-26

**Authors:** Jaemin Min, Hojun Lee, Moon Jeong Park

**Affiliations:** ^1^ Department of Chemistry Pohang University of Science and Technology (POSTECH) Pohang 37673 Republic of Korea

**Keywords:** block copolymers, self‐assembly, superlattices, zwitterions, dielectric constant, mechanical properties

## Abstract

Superlattice structures, created by the periodic arrangement of two or more distinct materials, have attracted significant interest. This paper proposes a strategy for designing superlattice structures in soft materials with improved dielectric and mechanical properties. Two types of acid‐functionalized block copolymers and a series of zwitterions with varying cationic and anionic structures are synthesized. When the zwitterion anion possesses a localized charge and the cation exhibits weak interactions with the acid groups in the polymers, the zwitterions preferentially localize at the interfacial layers between the ionic and ionophobic domains. As the zwitterion concentration increases and ion–dipole interactions strengthen within the confined subdomains, the interfacial curvature of the superlattice transitions from lamellae to cylindrical morphology and further evolves to Frank‐Kasper phases. Notably, these morphological transitions occur despite the symmetrical volume fractions of the ionic phases. These superlattice structures and Frank‐Kasper phases exhibit thermal stability up to 150 °C, a high static dielectric constant of 25, and excellent mechanical properties including a modulus of 360 MPa, hardness of 16 MPa, stiffness of 772 N m^−1^, and energy dissipation index of 0.675 at 22 °C, making them highly promising candidates for advanced applications in soft electronics and memory devices.

## Graphical abstract

Superlattices formed by the co‐assembly of acid‐functionalized block copolymers and zwitterions exhibit tunable morphologies, transitioning from lamellae to cylinders to Frank‐Kasper phases. Zwitterion interfacial localization enhances dielectric constant, mechanical strength, and thermal stability, making these materials promising for soft electronics and memory devices.

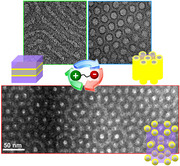

## 1 Introduction

Superlattice structures^[^ ^1^, ^2^ ^]^ characterized by the periodic arrangement of two or more distinct materials, inspired by the hierarchical structures and unique properties found in nature, such as those observed in abalones^[^ ^3^ ^]^ and insect eyes,^[^ ^4^ ^]^ have attracted significant interest. Mimicking such superlattice structures has often been achieved through layer‐by‐layer assembly of pre‐synthesized materials.^[^ ^5^, ^6^ ^]^ Another approach involves embedding or nucleating a material within preexisting nanostructured frameworks.^[^ ^7^, ^8^, ^9^, ^10^ ^]^ For example, metal‐polymer superlattices can be synthesized by selectively localizing metal precursors (followed by reduction)^[^ ^7^, ^8^ ^]^ or incorporating surface‐functionalized metal nanoparticles into specific nanodomains of self‐assembled polymers.^[^ ^9^, ^10^ ^]^

As pre‐structured frameworks, multiblock copolymers present a promising approach, owing to their ability to self‐assemble into multilayered configurations.^[^ ^11^, ^12^ ^]^ Ideally, the sequence and thickness of the individual layers, along with the interfacial curvature, can be tuned by controlling the block sequencing and the molecular weight of each block. However, achieving these structures requires a delicate balance of segregation strength,^[^ ^13^, ^14^ ^]^ which is a challenge exacerbated by the incorporation of high‐dipole‐moment components into microphase‐separated domains.

The ongoing pursuit of advanced superlattice materials that exhibit superior mechanical,^[^ ^15^, ^16^ ^]^ optical,^[^ ^17^, ^18^ ^]^ catalytic,^[^ ^19^, ^20^ ^]^ and electronic properties^[^ ^21^, ^22^ ^]^ is driven by their potential to outperform conventional materials. The performance of superlattice‐structured materials is largely dictated by the spatial arrangement and thickness of subdomains.^[^ ^23^ ^]^ However, achieving a uniform layer thickness and homogeneous composition in these materials remains a significant challenge, limiting their full potential in metamaterial development.

Recent strategies have explored the introduction of charged moieties to the side chains of linear^[^ ^24^, ^25^, ^26^ ^]^ or bottlebrush block copolymers.^[^ ^14^, ^27^, ^28^, ^29^ ^]^ Strong electrostatic interactions between these charged groups facilitate the formation of ionic subdomains within the microphase‐separated morphologies. However, precisely controlling the superlattice curvature by adjusting the concentration of charged groups and exploring advanced material properties through controlled molecular interactions remain largely unexplored.

In this study, a methodology for developing superlattices through the spontaneous coassembly of zwitterions and charged block copolymers is proposed. Unlike conventional lamellar (lam) or hexagonal cylindrical (hex) structures observed in block copolymers, the proposed methodology exploits the selective localization of zwitterions at the interfacial sublayers between the ionic and ionophobic domains. Through the investigation of the effect of molecular interactions and dipole arrangements of zwitterions within charged polymers on superlattice stabilization, a strategy to improve the dielectric properties and mechanical strength of hybrid materials has been explored. These materials offer a promising platform for next‐generation solvent‐free dielectric soft materials in wearable electronics, memory devices, and biomimetic systems.

## 2 Results and Discussion

### 2.1 Rational Design and Coassembly of Charged Block Copolymers and Zwitterions

**Figure 1:**
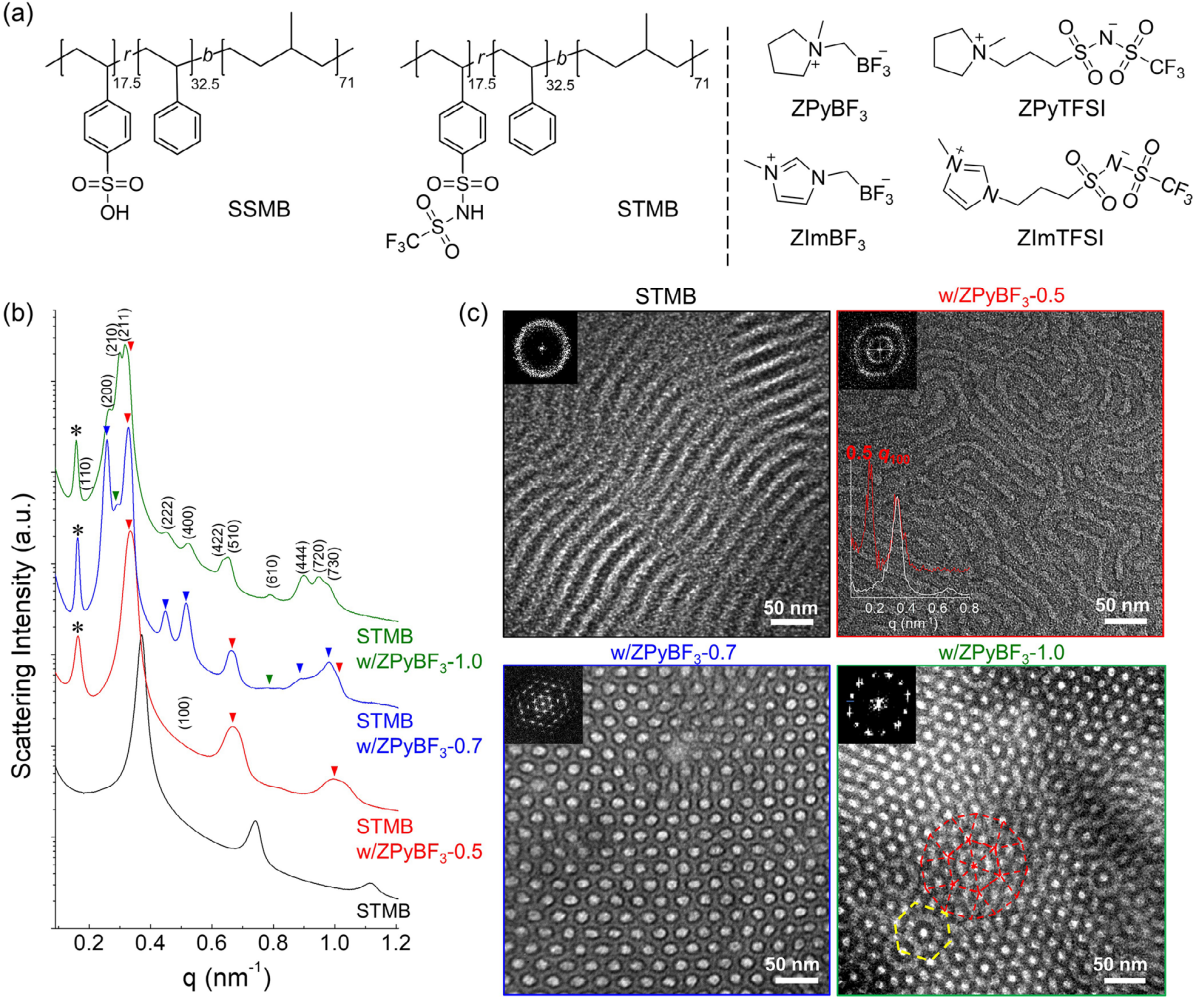
a) Chemical structures of acid‐functionalized block copolymers and zwitterions synthesized in this study. b) Small‐angle X‐ray scattering profiles (▼: lam; ▼: hex; ⁎: superlattice) and c) Bright‐field transmission electron microscopy micrographs with corresponding FFT patterns of poly(4‐styrenesulfonyl (trifluoromethanesulfonyl)imide‐*r*‐styrene)‐*b*‐polymethylbutylene (STMB) upon doping with ZPyBF_3_.

We designed two types of acid‐functionalized block copolymers using either sulfonic acid or sulfonylimide groups with identical degrees of polymerization, molecular weight distributions, and acid‐functional group sequences. Poly(4‐styrenesulfonic acid‐*r*‐styrene)‐*b*‐polymethylbutylene ([PSS‐*r*‐PS]‐*b*‐PMB, referred to as SSMB) was prepared by randomly sulfonating polystyrene‐*b*‐polymethylbutylene (5.0‐*b*‐5.0 kg mol^‐^¹) achieving a degree of sulfonation of 35 mol%. Subsequent imidization yielded poly(4‐styrenesulfonyl (trifluoromethanesulfonyl)imide‐*r*‐styrene)‐*b*‐polymethylbutylene ([PSTFSI‐*r*‐PS]‐*b*‐PMB, referred to as STMB). **Figure** **1**a shows the chemical structures of the synthesized polymers, with further synthetic details provided in the Experimental Section.

A series of zwitterions with varying cationic and anionic structures were synthesized to interact via ion‐dipole interactions with acid‐functional groups in polymers, as illustrated in Figure 1a, including trifluoro((1‐methylpyrrolidin‐1‐yl)methyl)borate (ZPyBF_3_), trifluoro((3‐methylimidazolium‐1‐yl)methyl)borate (ZImBF_3_), (3‐(1‐methylpyrrolidin‐1‐ium‐1‐yl)propyl)sulfonyl (trifluoromethyl)sulfonylimide (ZPyTFSI), and (3‐(3‐methyl imidazolium‐1‐yl)propyl)sulfonyl (trifluoromethyl)sulfonylimide (ZImTFSI) (Figures S1–S4, Supporting Information).

The Py^+^ and Im^+^ cations in the zwitterions were designed to exhibit varying binding strengths to the acid‐functional groups in the polymers, with Im^+^ cations exhibiting significantly stronger attractive interactions and shorter binding distances with the acid functional groups (Figure S5, Supporting Information). The ‐BF_3_^‐^ and ‒TFSI^‐^ anions were strategically selected to adjust the dipole moment and polarizability of zwitterions. For instance, the smaller ‒BF_3_^‐^ anions were anticipated to exhibit a localized charge distribution, resulting in higher dipole moments.

SSMB and STMB were doped with the zwitterions at different molar ratios relative to the acid‐functional groups in the polymers, and the resulting morphologies were investigated by combining small‐angle X‐ray scattering (SAXS) and transmission electron microscopy (TEM) experiments. Notably, the SSMB exhibited limited solvation capability and was miscible only with ZPyBF_3_ and ZImBF_3_. In contrast, STMB, containing delocalized and bulky TFSI groups, exhibited good miscibility with all four zwitterions: ZPyTFSI, ZImTFSI, ZPyBF_3_ and ZImBF_3_.

As shown in Figure 1b, the nominally symmetric STMB exhibits a lam structure with a domain size (*d*_100_ = 2π/*q*_100_) of 18.2 nm. The lam structures remained intact upon introducing 0.5 molar equivalents of ZPyBF_3_ per TFSI group (referred to as ZPyBF_3_‐0.5), whereas *d*_100_ increased by ≈10% (Bragg peaks are represented by inverted triangles). Notably, the addition of ZPyBF_3_ led to the appearance of a new peak at 0.5*q*_100_ (denoted as ⁎), indicating the formation of superlattice structures. Analogous lam superlattice structures could be observed at doping ratios of 0.1 and 0.3 (ZPyBF_3_‐0.1 and ZPyBF_3_‐0.3), with the SAXS profiles and isotropic 2D SAXS patterns remaining stable over a wide temperature range of 25 to 150 °C (Figure S6, Supporting Information).

Upon increasing the ZPyBF_3_ doping ratio to 0.7 (ZPyBF_3_‐0.7), coexisting hex phases emerged with a substantial increase in *d*_100_ to 24.3 nm. The SAXS profile exhibited a sharper and more intense superlattice peak at 0.5*q*_100_. In addition, a small peak appeared between the *q*_100_ peaks of the lam and hex structures, becoming more pronounced at an equimolar doping ratio (ZPyBF_3_‐1.0). The Bragg peaks could be indexed to A15 structures with a $Pm\bar{3}n$ space group and a lattice parameter of 48 nm, suggesting the formation of Frank‐Kasper (FK) phases with increasing ZPyBF_3_ content. This transition was accompanied by a notable reduction in the stability of the hex phases. The systematic phase transitions from lam‐to‐hex‐to‐FK phases, despite minimal zwitterion content variations, are particularly noteworthy.

The observed phase transitions were further examined by TEM, as shown in Figure 1c. RuO_4_ staining revealed that the nonionic PMB domains appeared as bright regions, whereas the PSTFSI‐*r*‐PS domains appeared dark. STMB doped with ZPyBF_3_‐0.5 exhibited lam superlattices, characterized by a high‐electron‐density interfacial layer (black) between the PMB (white) and PSTFSI (gray) domains. This result was in sharp contrast to the lam structure of neat STMB, which exhibited only two electron contrasts. A comparison of the Fast Fourier transform (FFT) of the TEM images for neat STMB and ZPyBF_3_‐0.5‐doped STMB clearly shows the formation of superlattice structures, as indicated by an additional ring pattern in the inner reciprocal space.^[^ ^30^ ^]^ The inset in the TEM image presents radial integrations of the 2D FFTs for both samples (with the white profile representing neat STMB and the red profile corresponding to ZPyBF_3_‐0.5‐doped STMB), confirming the presence of superlattices in ZPyBF_3_‐0.5‐doped STMB.

In ZPyBF_3_‐0.7, PMB cylinders encased by high‐electron‐density interfacial layers were arranged in a hex packing within the PSTFSI matrix, indicating the formation of hex superlattices. Upon increasing the doping to ZPyBF_3_‐1.0, the TEM image and diffraction pattern revealed phase transitions from hex structures to dodecagonal quasicrystals (DDQCs, marked by red dotted lines)^[^ ^31^ ^]^ or A15 structures (denoted by yellow dotted lines)^[^ ^32^ ^]^ via epitaxial routes, i.e., [001]_hex_ ↔ [111]_A15_. Although the underlying mechanism governing the formation of FK phases and DDQCs over body‐centered cubic lattices remains elusive, this transition should be driven by intensified molecular interactions within the interfacial sublayers.

**Figure 2:**
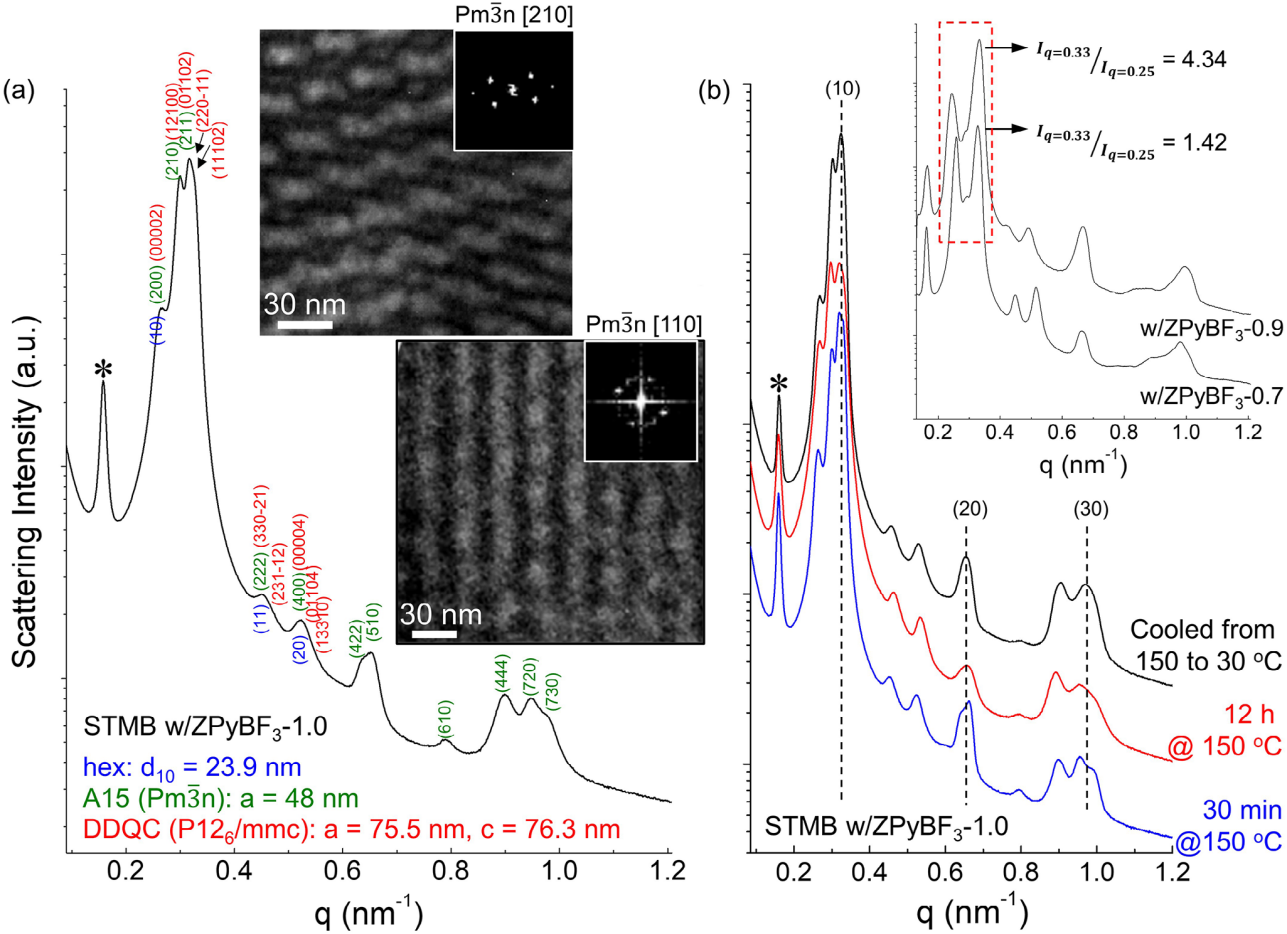
a) SAXS profile of STMB doped with ZPyBF_3_‐1.0, indexed to reflect the coexistence of hex, A15, and DDQC phases (⁎: superlattice). TEM images with corresponding FFT patterns show the A15 phase along the [110] and [210] projections. b) SAXS profiles of STMB doped with ZPyBF_3_‐1.0 at varying temperatures, with dotted vertical lines indicating the presence of coexisting lam superlattices. The inset SAXS profiles of STMB doped with ZPyBF_3_‐0.7 and ZPyBF_3_‐0.9 highlight the emergence of the A15 phase as the hex structure becomes destabilized.

The SAXS profile of ZPyBF_3_‐1.0‐doped STMB was further analyzed by coexisting A15, DDQC, and hex structures. For the DDQC phase assignment, Miller indices derived from the 5D space group symmetry P12_6_/mmc were employed. As shown in **Figure** **2**a, the structure factors of the hex, A15, and DDQC structures largely overlap in the low‐q region (q < 0.55 nm^−1^). Scattering peaks observed at q > 0.6 nm^−1^ could not be indexed using the 5D space group P12_6_/mmc, but were fully assignable using the $Pm\bar{3}n$ space group. We thus conclude that the predominant phase in STMB doped with ZPyBF_3_‐1.0 is the FK structures, with minor contributions from DDQCs and persistent lam superlattices. Furthermore, extended‐area TEM imaging was performed, revealing structural projections characteristic of the A15 structures along the [110] and [210] directions, as shown in the insets of Figure 2a. Regions exhibiting DDQCs are presented in Figure S7 (Supporting Information). It is noteworthy that A15 phases share local structural motifs with DDQCs, complicating the identification of their respective contributions in ZPyBF_3_‐1.0‐doped STMB.

It is important to note that the coexistence of lam superlattices and FK phases was maintained even at 150 °C. To assess the thermal stability of the coexisting morphologies in STMB with ZPyBF_3_‐1.0, in situ SAXS measurements were conducted during heating from 30 to 150 °C, followed by an isothermal hold at 150 °C, as shown in Figure 2b. After 30 min at 150 °C, the coexistence of A15 structures and lam superlattices was evident. With extended annealing at 150 °C for 12 h, the intensity of the lam peaks gradually decreased, accompanied by an increase in the (200) and (210) peaks of the A15 structures. Further annealing for 24 h at 150 °C resulted in no further noticeable changes in the SAXS profile. Upon cooling the sample back to 30 °C, the lam peaks rapidly re‐emerged. These results suggest that the FK phases are thermodynamically favored at elevated temperatures, and the lam‐to‐A15 transition proceeds slowly due to the need for interfacial splitting and reorganization into spherical domains. In contrast, the A15‐to‐lam transition upon cooling is rapid.

Next, the development of FK phases with increasing ZPyBF_3_ concentration was examined. To this end, we investigated the morphology of STMB doped with 0.9 equivalents of ZPyBF_3_. As shown in the inset SAXS profiles of Figure 2b, increasing the ZPyBF_3_ concentration from 0.7 to 0.9 equivalents led to a marked reduction in the (10) peak intensity of the hex structures, along with an increase in the (211) peak intensity of the A15 phase. This shift is highlighted by a threefold increase in the intensity ratio of I_211, A15_ (q = 0.33 nm^−1^) to I_10, hex_ (q = 0.25 nm^−1^). Additionally, the (11) and (20) peaks of the hex structures, along with the superlattice peak, were also weakened. These changes point to a gradual structural transformation toward FK phases as the ZPyBF_3_ loading increases, accompanied by a concurrent destabilization of the hex morphology.

### 2.2 Molecular Interactions and Dipolar Arrangements of Zwitterions within Charged Polymers

**Figure 3:**
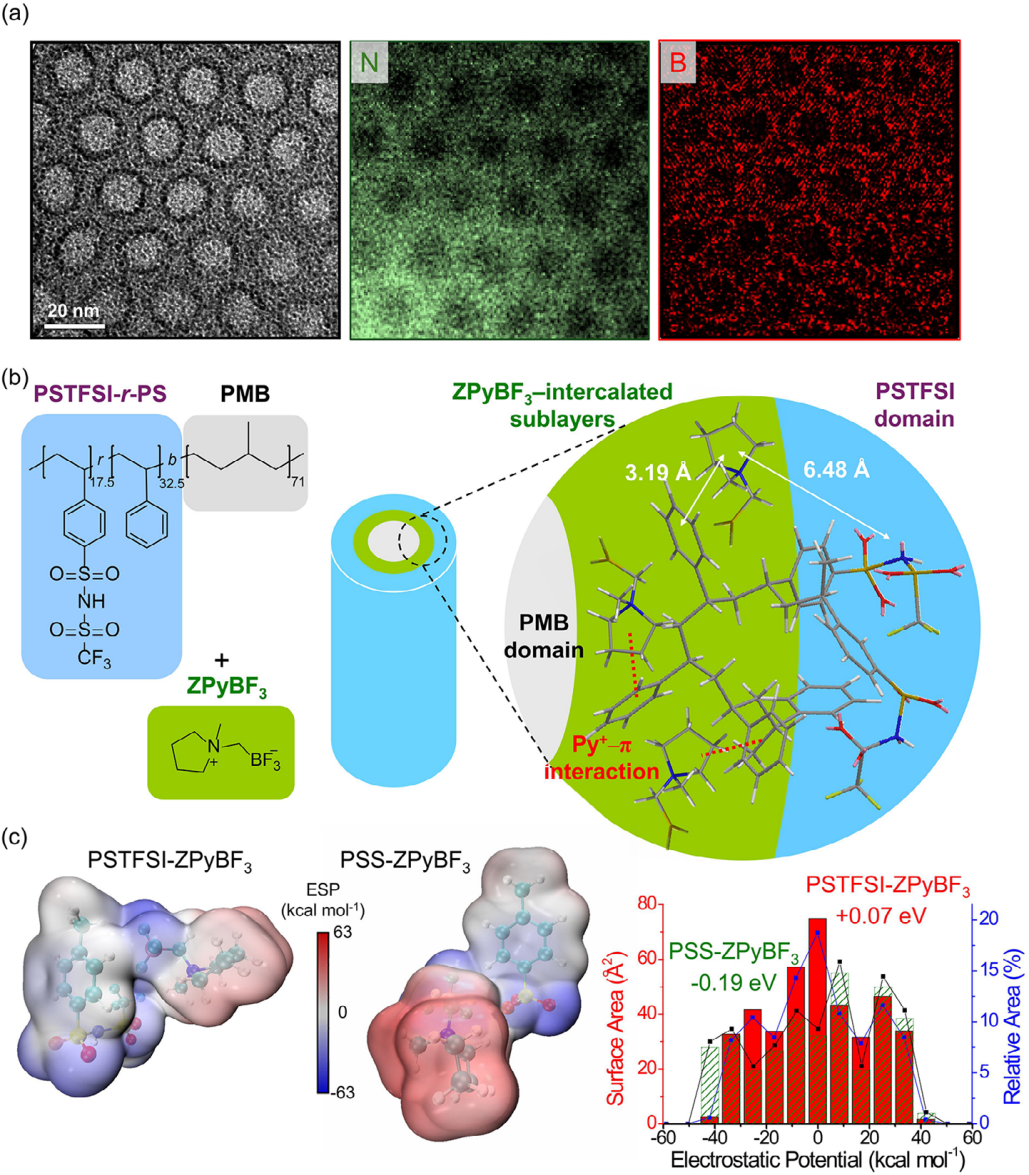
a) Bright‐field TEM image and elemental mapping images of sulfur and fluorine atoms for ZPyBF_3_‐0.7‐doped STMB. b) Schematic showing distinct positioning of ZPyBF_3_ within sublayers of self‐assembled STMB, leading to the formation of superlattice structures. c) DFT‐optimized molecular configurations and electrostatic potential (ESP) map analyses of PSTFSI and PSS monomeric units in interaction with ZPyBF_3_. In the plot on the right, the red histogram represents PSTFSI, while the green dashed histogram corresponds to PSS.

To investigate the nature of the high‐electron‐contrast interfacial sublayers, we performed TEM elemental mapping of the nitrogen and boron atoms, as shown in **Figure** **3**a. Boron mapping selectively visualizes the location of ZPyBF_3_, whereas nitrogen mapping highlights PSTFSI domains. By comparing these two elemental maps, we inferred that the high‐electron‐contrast interfacial sublayers originate from ZPyBF_3_ enrichment. This enrichment created distinct interfaces between the nonionic PMB and the ionic PSTFSI domains, as evidenced by the bright rings in the boron‐mapped images.

The co‐assembly and molecular arrangement of ZPyBF_3_ in the STMB superlattice are schematically depicted in Figure 3b. The selective localization of ZPyBF_3_ at the interfaces effectively reduced interfacial energies by concentrating the small ‒BF_3_^‐^ anions. The observed lam‐to‐hex phase transition with increasing ZPyBF_3_ content cannot be attributed to ionic domain swelling because the calculated volume fraction of the ionic components increased only marginally from 0.55 to 0.57 (**Table** **1**). Instead, the enhanced ion–ion and ion–dipole interactions within the confined passivation layers with increasing ZPyBF_3_ content were responsible for the greater interfacial curvature.

**Table 1:** ZPyBF_3_‐doped STMB samples at different molar ratios.

Sample code [STMB w/]	The molar ratio of ZPyBF_3_ to ‐TFSI units in STMB	Mass fraction of ZPyBF_3_ [wt.%]	Volume fraction of ionic phase [*f*_PSTFSI_+ *f*_ZPyBF3_]^a)^
ZPyBF_3_‐0.1	0.1	2.0	0.52
ZPyBF_3_‐0.3	0.3	5.8	0.54
ZPyBF_3_‐0.5	0.5	9.4	0.55
ZPyBF_3_‐0.7	0.7	13	0.56
ZPyBF_3_‐1.0	1.0	17	0.57

^a)^ calculated using constituent densities and van der Waals volumes computed by Chemaxon.

Density functional theory (DFT)^[^ ^33^, ^34^ ^]^ calculations indicated that ZPyBF_3_ does not exhibit attractive interactions with the PSTFSI unit by showing a binding energy of +0.07 eV (Figure S5, Supporting Information). Therefore, we propose that it preferentially associates with the PS chain through Py^+^‒ π interactions to facilitate the formation of a ZPyBF_3_‐PS passivation layer at the PSTFSI‐*r*‐PS/PMB interface. This hypothesis is further supported by DFT calculations which show that the insertion of a PS unit into the PSTFSI segment lowers the binding energy with ZPyBF_3_ to −0.4 eV. The DFT‐optimized geometries of PSTFSI‐*r*‐PS with ZPyBF_3_ are presented in Figure 3b.

Electrostatic potential (ESP) map analyses^[^ ^35^, ^36^ ^]^ revealed pronounced differences in the charge distribution of ZPyBF_3_ when interacting with PSTFSI compared to PSS, with the former exhibiting a markedly lower ESP gap. As shown in Figure 3c, the ESP profiles of PSTFSI/ZPyBF_3_ (red histogram) and PSS/ZPyBF_3_ (green dashed histogram) highlight this contrast. It should be noted here that ZImBF_3_ led to highly polarized ESP values in both PSTFSI and PSS samples (Figure S8, Supporting Information), resulting in its uniform distribution within ionic domains— a behavior commonly reported for zwitterion‐doped ionic polymers in literature.^[^ ^37^ ^]^

**Figure 4:**
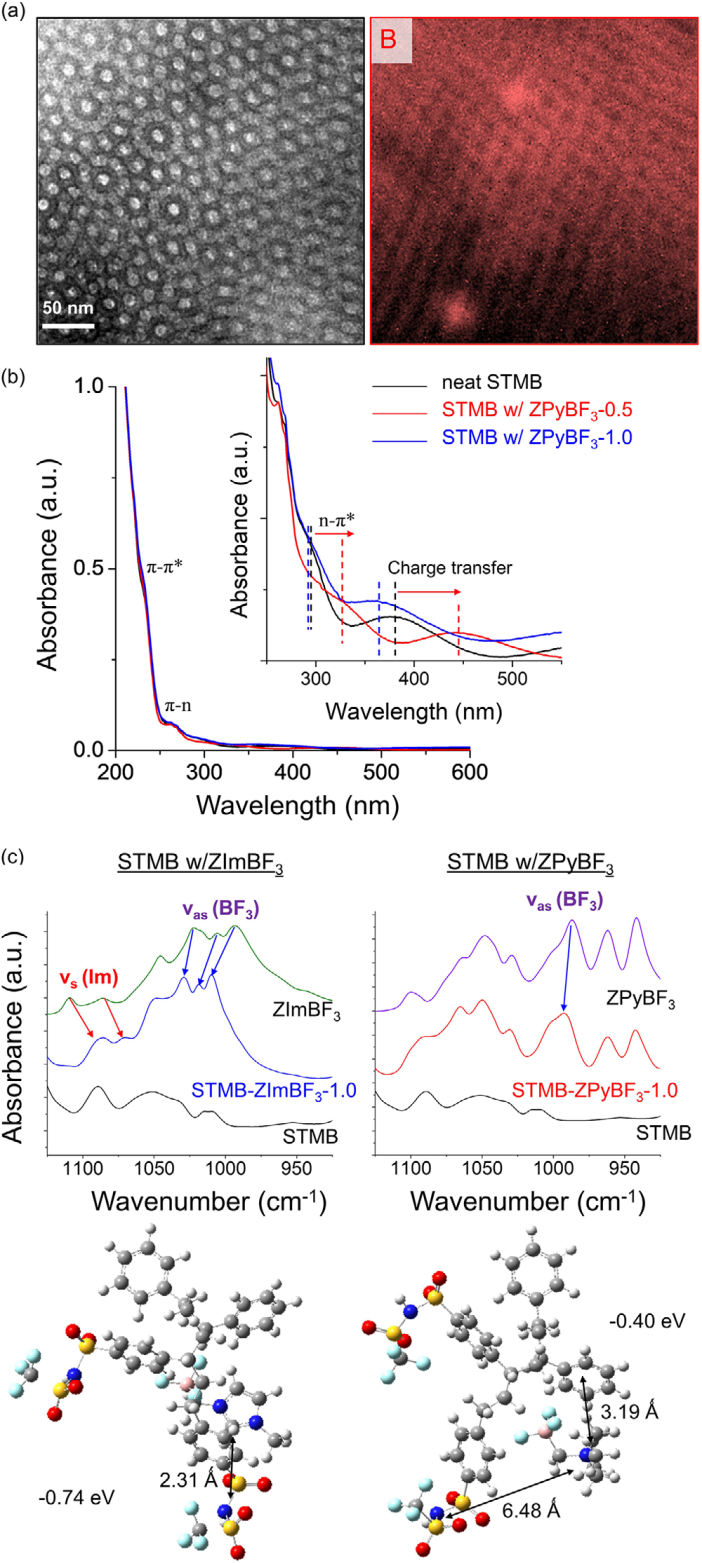
a) Bright‐field TEM image and boron mapping image for ZPyBF_3_‐1.0‐doped STMB. b) UV–vis spectra of STMB samples doped with ZPyBF_3_, showing considerable changes in the n→π* charge transfer band as a function of doping level. c) FT‐IR spectra of STMB doped with ZImBF_3_ and ZPyBF_3_. DFT‐optimized molecular configurations illustrating the interactions between PSTFSI‐*r*‐PS units and each zwitterion are shown in the lower panel.

We also conducted boron mapping on STMB doped with ZPyBF_3_‐1.0 to investigate the location of zwitterions within the FK phases. However, due to the limited spatial resolution of TEM elemental mapping, obtaining clear boron maps in the samples with 3D spherical morphologies proved substantially more challenging than in 2D‐structured counterparts. As shown in Figure 4a, while boron signals were detected throughout the ionic matrix, their specific distribution across the interfaces of PMB/PS/PSTFSI could not be resolved.

The persistence of the superlattice SAXS peak in ZPyBF_3_‐1.0‐doped STMB suggests that the ZPyBF_3_‐enriched passivation layer retains its structural integrity, which is beyond the resolution of current TEM imaging. As zwitterion loading increases, the interfacial sublayers within the hex superlattices appear unable to fully accommodate the additional zwitterions. This promotes interfacial mixing between the ionic and non‐ionic domains, which relieves packing frustration and enables the formation of FK phases.^[^ ^38^ ^]^

Further insights are provided by UV–vis spectroscopy. As shown in Figure 4b, a pronounced redshift in the n→π* charge transfer band was observed upon doping with ZPyBF_3_‐0.5. This shift is attributed to interactions between the lone pair electrons of the zwitterion and the π* orbitals of the PS phenyl rings, which disrupt the ring–ring stacking.^[^ ^39^ ^]^ Such interactions are favored in lam or hex superlattices characterized by well‐defined interfacial ZPyBF_3_‐PS sublayers. Interestingly, upon the formation of FK phases at ZPyBF_3_‐1.0 loading, the charge transfer band exhibits a blue shift. This shift is attributed to the distribution of zwitterions throughout the PSTFSI matrix, where ion–dipole interactions withdraw electron density from the phenyl rings of PSTFSI.

The molecular interactions between STMB and the zwitterions were investigated by Fourier transform infrared spectroscopy. As shown in Figure 4c, incorporating ZImBF_3_ into STMB leads to pronounced redshifts in the in‐plane vibration peaks of the Im^+^ ring and a noticeable blue shift in the B─F asymmetric stretching vibration peaks. This blue shift can be attributed to the enhanced restoring force on the vibrating B─F bonds. In contrast, the characteristic peaks of ZPyBF_3_ exhibit minimal changes when embedded in STMB, suggesting weak ionic interactions between ZPyBF_3_ and ‐TFSI groups. This is further supported by the negligible shift in glass transition temperature of PSTFSI chains upon ZPyBF_3_ doping, despite the lam‐to‐hex phase transition. Additionally, the crystallization behavior of ZPyBF_3_ is entirely suppressed in STMB, even at high ZPyBF_3_ doping levels such as ZPyBF_3_‐1.6 (Figure S9, Supporting Information). This behavior is attributed to the disruption of the antiparallel molecular arrangements caused by the localization of ZPyBF_3_ at the interfacial subdomains. As further supported by DFT calculations, ZPyBF_3_ preferentially resides near the PS phenyl rings, whereas ZImBF_3_, exhibits a stronger affinity for the PSTFSI segments.

It is worth noting that unique superlattice structures and FK phases were observed exclusively in the ZPyBF_3_‐doped STMB samples. As shown in Figure S10 (Supporting Information), ZImBF_3_‐0.5 induced hex structures in STMB through effective swelling of PSTFSI domains, whereas ZPyTFSI or ZImTFSI led to less ordered lam structures. Replacing zwitterions with neutral imidazole (Im) preserved the lam structure in STMB with only a small increase in the domain size without any indication of superlattice structures. For the zwitterion‐doped SSMB samples, only conventional lam or hex morphologies were observed, with less swollen ionic domains compared to their STMB counterparts.

These observations highlight that the zwitterions and the selection of acid functional groups in the polymer are critical in controlling the localization and directing ion–dipole arrangements within the subdomains of charged block copolymers by precisely tuning their molecular interactions. Moreover, the strong binding of additives to acid functional groups (e.g., Im^+^‒TFSI^‒^ or zwitterion‒SO_3_H) restricts their localization at the interfacial sublayers.

### 2.3 Enhancement of Dielectric and Mechanical Properties of Charged Polymers

**Figure 5:**
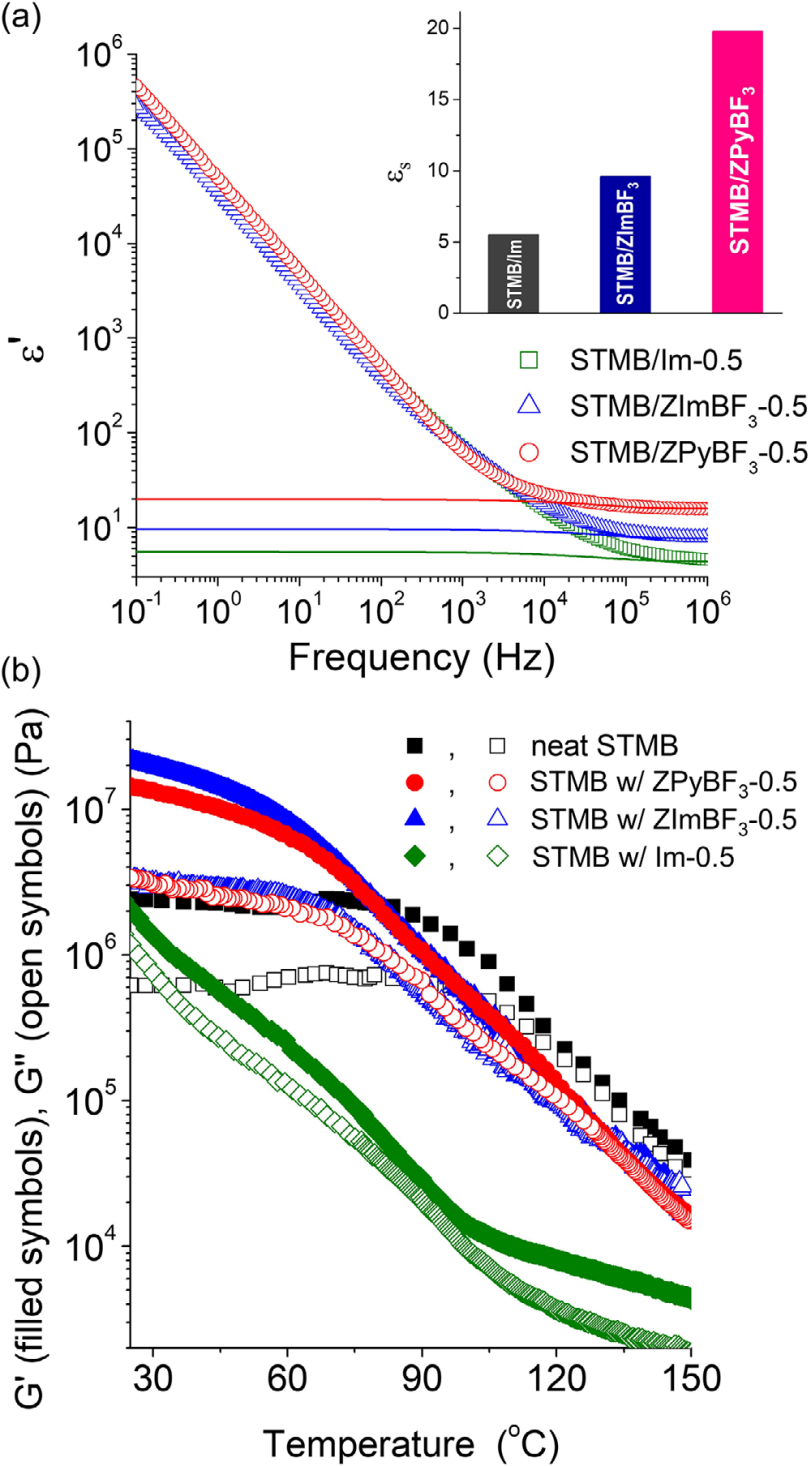
a) Dielectric permittivity spectra of STMB doped with Im‐0.5, ZImBF_3_‐0.5, or ZPyBF_3_‐0.5, measured at 25 °C. Static dielectric constants obtained by model fits are shown in the inset. b) Storage (G′) and loss moduli (G″) of STMB upon doping with Im or zwitterions in the range of 25 and 150 °C.

Motivated by the high static dielectric constants (ε_s_) of zwitterions, typically exceeding 30 and strongly dependent on their chemical structures,^[^ ^40^, ^41^ ^]^ we investigated the dielectric relaxation behavior of STMB upon zwitterion incorporation. Representative dielectric permittivity spectra of STMB doped with ZPyBF_3_, ZImBF_3_, or Im, measured at 25 °C, are shown in **Figure** **5**a. The doping level of all the additives was maintained at half equimolar (e.g., ZPyBF_3_‐0.5). Given the low dielectric constant (ε_s_ < 5) of STMB, comparable to those of most polymers, the incorporation of ≈9 wt.% zwitterion was not expected to significantly enhance the ε_s_ of the system.

The ε_s_ values were extracted from fitted lines,^[^ ^42^ ^]^ as illustrated in Figure 5a, with the corresponding values summarized in the inset plot (details are presented in Figure S11, Supporting Information). Notably, STMB doped with ZPyBF_3_ exhibited a significantly high ε_s_ value of 20, whereas incorporation of ZImBF_3_ yielded a much lower ε_s_ of 9.6, which was less than half that of the ZPyBF_3_‐doped system. The lowest ε_s_ value of 5.5 was observed for the Im‐doped STMB. These low ε_s_ can be attributed to the small molecular dipole moment of Im (3.7 D), as estimated by the DFT calculations, compared to 10.6 D of ZPyBF_3_ and 12.0 D of ZImBF_3_. However, the higher ε_s_ value of the ZPyBF_3_‐doped sample, greater than twice that of the ZImBF_3_‐doped sample, cannot be solely attributed to differences in molecular polarity. When ethylene glycol (EG) (ε_s_ = 37 at 20 °C) was used to dissolve zwitterions, adding ∼10 wt.% significantly increased ε_s_ to 50.3 for ZPyBF_3_/EG and 64.8 for ZImBF_3_/EG (Figure S12, Supporting Information), consistent with literature.^[^ ^41^ ^]^ The higher ε_s_ value of ZImBF_3_/EG aligns with its greater molecular dipole moment.

We propose that the substantial increase in ε_s_ for ZPyBF_3_‐doped STMB arises from the intercalation of ZPyBF_3_ into the interfacial PS sublayers, as supported by the DFT calculations indicating unfavorable interactions between ZPyBF_3_ and the PSTFSI chain. These layers disrupt quadrupole formation, enabling the surrounding dipoles to align in a parallel direction, resulting in the generation of a net dipole moment. This net dipole moment is closely related to the tight packing of neighboring dipolar moieties, facilitating dipole–dipole interactions that contribute enthalpically to subdomain formation. These interactions stabilize the superlattice structures by forming ZPyBF_3_‐rich sublayers. This mesoscopic ordering likely promotes enhanced dipolar alignment and collective polarization under an external electric field, resulting in substantially increased ε_s_. In contrast, Im and ZImBF_3_ appeared to be molecularly dispersed within the PSTFSI domains because of their favorable interactions within the TFSI groups. These interactions led to the formation of either ion pairs (TFSI^‐^–Im^+^) or quadrupoles, where antiparallel dipole arrangements result in mutual dipole cancellation.

Notably, as shown in Figure 5b, zwitterion doping significantly enhanced the mechanical properties of STMB. Incorporating ZPyBF_3_‐0.5 led to a six‐fold increase in the shear moduli at room temperature, with a storage modulus (G′) of 15 MPa and loss modulus (G′) of 3 MPa. Doping with ZImBF_3_‐0.5, further increased the modulus to G′ = 23 and G″ = 3 MPa. The dissimilar moduli were, in part, attributed to their distinct morphologies, with ZPyBF_3_‐0.5 adopting a lam structure, and ZImBF_3_ forming a hex structure. Importantly, both zwitterion‐doped STMB samples maintained their elastic behavior and high shear modulus (>1 MPa) even at 90 °C, attributed to the preserved glassy nature of PSTFSI combined with molecular ion–dipole interactions that served as dense physical crosslinks. In contrast, Im‐doping caused plasticization of the PSTFSI chains, leading to a substantial decrease in the moduli and inducing rubbery behavior over the entire temperature range studied.

**Figure 6:**
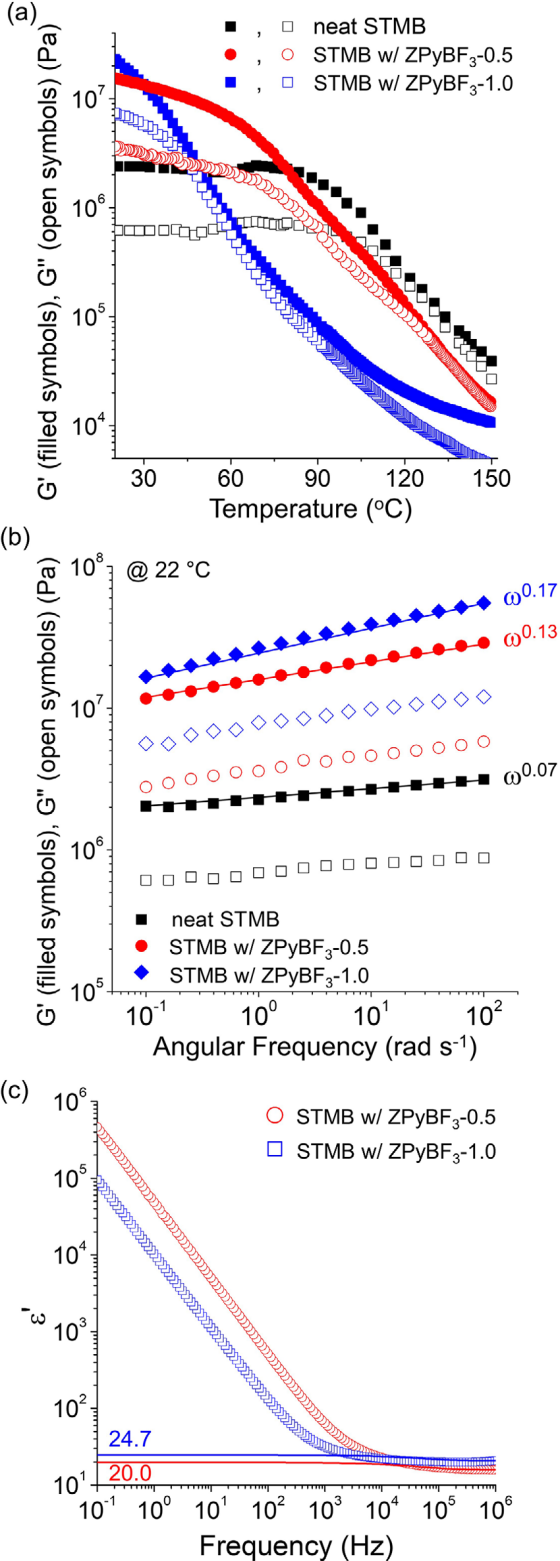
Storage (G′) and loss moduli (G″) of STMB upon doping with ZPyBF_3_‐0.5 or ZPyBF_3_‐1.0; a) temperature sweep in the range of 25 and 150 °C; b) frequency sweep from 0.1 to 100 rad s^−1^. c) Dielectric permittivity spectra of STMB doped with ZPyBF_3_‐0.5 or ZPyBF_3_‐1.0, measured at 25 °C. Static dielectric constants obtained by model fits are shown in the figure.

As shown in **Figure** **6**a, increasing the ZPyBF_3_ loading from 0.5 to 1.0 equivalents in STMB samples led to a substantial enhancement in mechanical properties, with the G′ rising to 22 MPa and the G″ to 7 MPa at 20 °C. This improvement is attributed to a higher density of physical cross‐links formed via ion–dipole interactions. The morphological transition from lam to 3D FK phases also played a role in this enhancement. However, for ZPyBF_3_‐1.0, the moduli dropped significantly with increasing temperature, which is attributed to the increased plasticization of the PSTFSI chains with zwitterions and more extensive interfacial mixing between the glassy PSTFSI‐*r*‐PS and the rubbery PMB blocks.

To further elucidate the mechanism of the enhancement of mechanical properties with zwitterion addition, we measured frequency‐dependent linear viscoelastic properties at different ZPyBF_3_ concentrations at 20 °C (< T_g_). The G′ and G″ gradually increase with increasing frequency, where the less ZPyBF_3_ doping less frequency dependence, suggesting a more solid‐like structure. The storage moduli exhibited increasing power law exponents, G′≈ω^0.07^ (neat STMB), ω^0.13^ (ZPyBF_3_‐0.5), and ω^0.17^(ZPyBF_3_‐1.0), as shown in Figure 6b. Despite this variation, all three samples showed weak frequency dependence for both G′ and G″, with G′ > G″, characteristic of hard gel behavior in both lam superlattices and FK phases.

The ε_s_ value of STMB samples continues to increase with higher zwitterion content. As shown in Figure 6c, the ε_s_ value reaches 24.7 in STMB doped with ZPyBF_3_‐1.0. Compared to zwitterion‐doped EG systems, the increase in ε_s_ in STMB samples is modest due to the restricted rotational mobility of zwitterions within the polymer matrix and the overlap of polarizability volumes. In contrast, low‐viscosity environments of small molecules allow greater orientational freedom to zwitterions.^[^ ^43^, ^44^ ^]^ Nevertheless, the simultaneous enhancement of both moduli and ε_s_ values in the zwitterion‐doped STMB, achieved through the formation of FK structures, represents a significant advancement.

**Figure 7:**
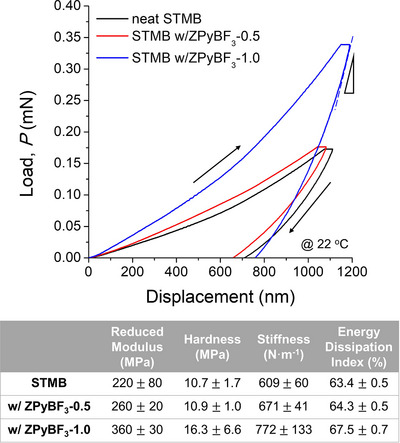
Load/unload curves of STMB, STMB with ZPyBF_3_‐0.5, and STMB with ZPyBF_3_‐1.0, acquired through depth‐sensing nanoindentation. The table summarizes the corresponding values for modulus, hardness, stiffness, and energy dissipation index.

The mechanical robustness of the STMB samples was further evaluated using depth‐sensing nanoindentation. The samples had an approximate thickness of 170 µm, and a Poisson's ratio of 0.35, corresponding to glassy polystyrene, was used in the displacement‐to‐area calculations.^[^ ^45^ ^]^ **Figure** **7** presents the loading and unloading curves for neat STMB, STMB with ZPyBF_3_‐0.5, and STMB with ZPyBF_3_‐1.0. Corresponding mechanical properties derived from the Oliver–Pharr analysis are summarized in the accompanying table.

As shown, the reduced modulus (E) of neat STMB was 220 MPa, increasing to 260 MPa with ZPyBF_3_‐0.5 and further to 360 MPa with ZPyBF_3_‐1.0. Similar trends were observed for hardness (H) and stiffness (S), with the increases becoming more pronounced upon the addition of ZPyBF_3_‐1.0, demonstrating the beneficial role of the FK phases in enhancing mechanical properties compared to the lam (ZPyBF_3_‐0.5) structures. In other words, the development of dynamic physical cross‐links, enabled by the embedded zwitterions within the interconnected ionic matrix of the FK structures, enhanced resistance to mechanical deformation. Although the modulus increased substantially with ZPyBF_3_ loading, the energy dissipation index showed only modest changes, and the highest value of 67.5% was observed for ZPyBF_3_‐1.0‐doped STMB. This indicates that, despite the sample displaying enhanced stiffness due to FK phase formation, the toughening mechanism is primarily governed by shear banding or viscoelastic energy dissipation.

These findings indicate that the zwitterion‐induced ion–dipole interactions play a critical role in enhancing the mechanical properties of charged polymers. Remarkably, simultaneous improvement in the dielectric and mechanical properties of the charged block copolymers is achievable through the formation of zwitterion‐localized subdomains, leading to a fully solvent‐free and non‐volatile solid‐state design. This result is particularly compelling because the traditional methods for controlling the nanoscale morphologies of block copolymers, such as lam, hex, and spherical structures, usually depend on the selective incorporation of additives into one of the blocks,^[^ ^46^, ^47^, ^48^ ^]^ often resulting in a trade‐off between these properties.^[^ ^49^, ^50^ ^]^

## 3 Conclusion

This study presents a straightforward strategy for enhancing the dielectric and mechanical properties of soft materials through the co‐assembly of acid‐functionalized block copolymers and zwitterions with tailored molecular interactions. This approach is based on the passivation of zwitterions at the interfaces between the ionic and ionophobic domains. By increasing the zwitterion concentration while maintaining a symmetric block composition, the interfacial curvature of the superlattices can be precisely controlled from lam‐to‐hex‐to‐FK phases, driven by strengthened ion–dipole interactions within the confined interfacial layers. The stabilization of superlattices was governed by three key principles: 1) partial charging of the ionic polymers to reinforce the passivation layer, 2) the use of additives with permanent dipoles and localized charges to direct molecular organization within subdomains by lowering the interfacial energy, and 3) weak interactions between zwitterions and ionic polymers to inhibit their uniform distribution throughout the ionic domain. This approach offers a promising platform for next‐generation dielectric soft materials in wearable electronics, memory devices, and biomimetic systems.

## Conflict of Interest

The authors declare no conflict of interest.

## Supporting information



Supporting Information

## Data Availability

The data that support the findings of this study are available in the Supporting Information of this article.
