# Supplementary material for: Zwitterion‐Stabilized Superlattices and Frank‐Kasper Phases in Charged Block Copolymers for Mechanically Robust Dielectric Soft Materials
Source: Adv Sci (Weinh). 2025 Jun 26;12(36):e07115. doi: 10.1002/advs.202507115 (PMC12463121; doi:10.1002/advs.202507115)
Supplement: Supplementary file 1 — Supporting Information [file ADVS-12-e07115-s001.docx]

Supporting Information

Zwitterion-Stabilized Superlattices and Frank-Kasper Phases in Charged Block Copolymers for Mechanically Robust Dielectric Soft Materials

*Jaemin Min*^§^*, Hojun Lee*^§^*,* *and Moon Jeong Park**

**Experimental Section**

**Materials**

Styrene (≥ 99%), isoprene (≥ 99%), sec-butyllithium (1.4M in cyclohexane), cyclohexane (≥ 99.5%, anhydrous), p-toluenesulfonylhydrazide (≥ 97%), acetic anhydride (≥ 99.5%), sulfuric acid (≥ 99.99%), 1,2-dichloroethane (≥ 99.8%, anhydrous), tetrahydrofuran (THF, ≥ 99.9%, anhydrous), methanol (≥ 99.9, anhydrous), N,N-dimethylformamide (DMF, ≥ 99.8%, anhydrous), 4-dimethylaminopyridine (DMAP, ≥ 99%), triethylamine (TEA, ≥ 99.5%), dichloromethane (≥ 99.8%), sodium bicarbonate (≥ 99.9%), methanol (≥ 99.9%, anhydrous), dimethylsulfoxide (DMSO, ≥ 99.9%, anhydrous), 3-chloro-1-propanesulfonyl chloride (≥ 98.0%), 1-methylpyrrolidine (≥ 98.0%), 1-methylimidazole (≥ 99.0%), acetone (≥ 99.5%), and ethylene glycol (anhydrous, ≥ 99.8%) were purchased from Sigma-Aldrich. Oxalyl chloride (≥ 98.0%), hydrochloric acid (HCl, 1M), trifluoromethanesulfonamide (≥ 98.0%), potassium carbonate (≥ 99.0%), potassium bromomethyltrifluoroborate (≥ 98.0%), and o-Xylene (≥ 98.5%) were obtained from TCI.

**Synthesis of acid-functionalized block copolymers**

A polystyrene-*b*-polymethylbutylene (PS-*b*-PMB) block copolymer was synthesized following the procedure described in ref. [1]. The number-averaged molecular weight (*M*_n_) of PS-*b*-PMB was determined to be 5.0-*b*-5.0 kg mol^-1^ using end-group analysis via ^1^H nuclear magnetic resonance spectroscopy (^1^H-NMR, Bruker AVB-500). Size exclusion chromatography (SEC) analysis, performed using a Waters Breeze 2 HPLC with THF as the eluent, confirmed a molecular weight distribution of 1.05. Poly(4-styrenesulfonic acid-*r*-styrene)-*b*-polymethylbutylene ([PSS-*r*-PS]-*b*-PMB, SSMB) was synthesized by randomly sulfonating PS-*b*-PMB with acetyl sulfate in dichloroethane at 25 °C for 50 min, achieving a sulfonation level of 35 mol%. To obtain poly(4-styrenesulfonyl(trifluoromethanesulfonyl)imide-*r*-styrene)-*b*-polymethylbutylene ([PSTFSI-*r*-PS]-*b*-PMB, STMB) with the identical degree of polymerization, molecular weight distribution, and acid-functional group sequence as SSMB, a two-step modification of SSMB—chlorination followed by imidization—was conducted at 25 °C for one day per step within an argon-filled glovebox.^[26]^ The successful completion of each chlorination and imidization step was confirmed through a combination of Fourier transform infrared (FT-IR) spectroscopy (Spectrum Two FT-IR, PerkinElmer) and ^19^F-NMR (Bruker AVB-300). SSMB and STMB were purified through dialysis against distilled water, followed by precipitation and freeze drying for two weeks.

**Synthesis of zwitterions**

Trifluoro((1-methylpyrrolidin-1-ium-1-yl)methyl)borate (ZPyBF_3_): ZPyBF_3_ was synthesized by procedure described in ref. [2]. Potassium bromomethyltrifluoroborate (1g, 4.97 mmol) was dissolved in acetone (20 mL) at 45 °C, and 1-methylpyrrolidine (0.8 g, 9.40 mmol) was added dropwise into the solution. The mixture was refluxed for 1 day at 45 °C. The resulting crude product was dissolved in methanol and subjected to recrystallization at −30 °C, followed by filtration and washing with cold methanol. Subsequent vacuum drying yielded ZPyBF_3_ (0.54g, 65% yield). ^1^H NMR (500 MHz, D_2_O) δ_H_ (ppm): 3.3–3.4 (m, 4H, N(C**H**_2_)_2_), 2.93 (s, 3H, N(C**H**_3_)), 2.43 ppm (m, 2H, N(C**H**_2_)BF_3_) and 2.07 ppm (m, 4H, NCH_2_(C**H**_2_)_2_). ^19^F NMR (470 MHz, D_2_O) δ_F_ (ppm): −159.8, −159.9, −160.1, −160.2


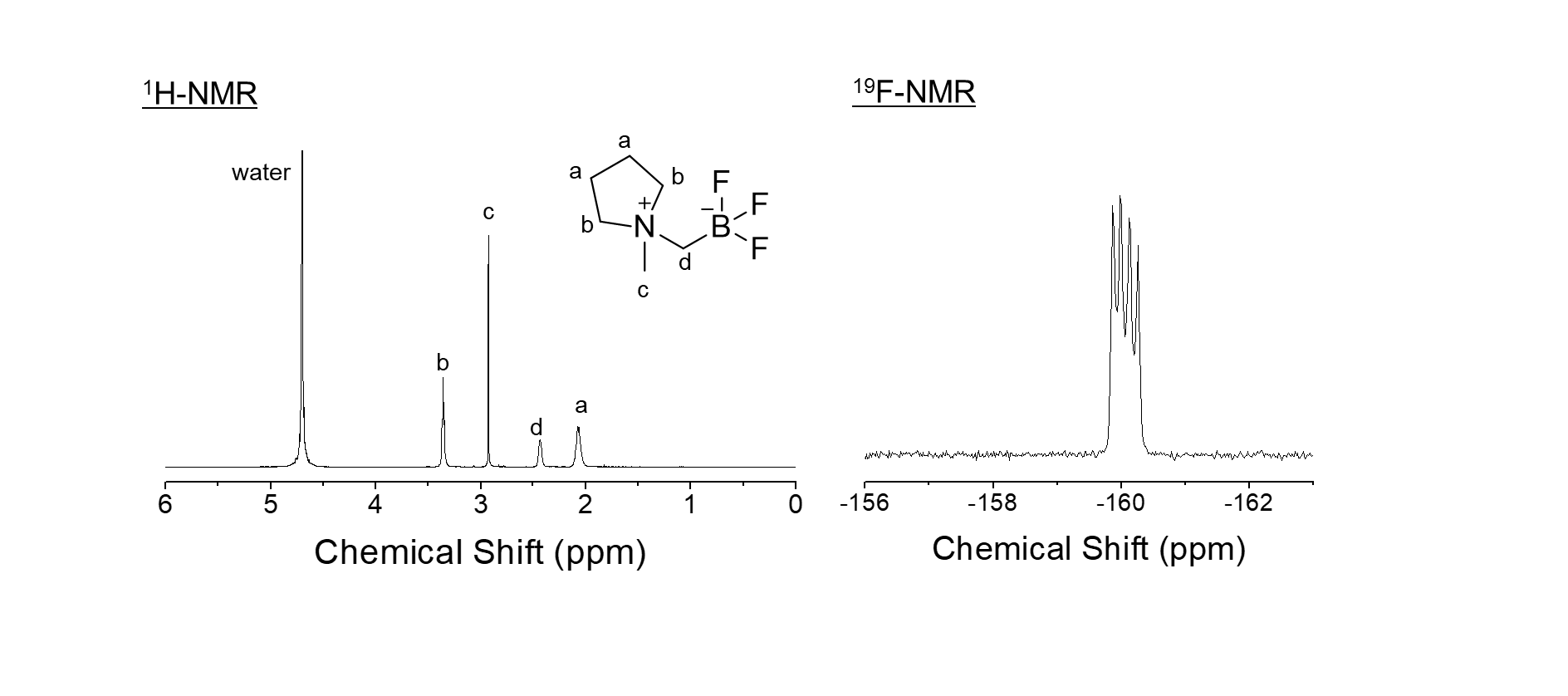


**Figure S1.** ^1^H-NMR and ^19^F-NMR spectra of ZPyBF_3_.

Trifluoro((3-methylimidazolium-1-yl)methyl)borate (ZImBF_3_): Potassium bromomethyltrifluoroborate (1 g, 4.97 mmol) was dissolved in 1-methylimidazole (20 mL) and refluxed at 130 °C for 48 h. The mixture was dissolved in methanol/water cosolvent and refluxed for 15 min. The solution was then cooled down to −30 °C for recrystallization, filtered, and the resulting solid was rinsed with cold methanol. Subsequent vacuum drying yielded ZImBF_3_ (0.42 g, 52% yield). ^1^H NMR (500 MHz, D_2_O) δ_H_ (ppm): 8.38 (s, 1H, N(C**H**)N), 7.25, (m, 2H, N(C**H**C**H**)N), 3.79 (s, 3H, N(C**H**_3_)), 3.27 (m, 2H, N(C**H**_2_)BF_3_), ^19^F NMR (470 MHz, D_2_O) δ_F_ (ppm): −142.7, −142.8, −142.9, −143.0

**
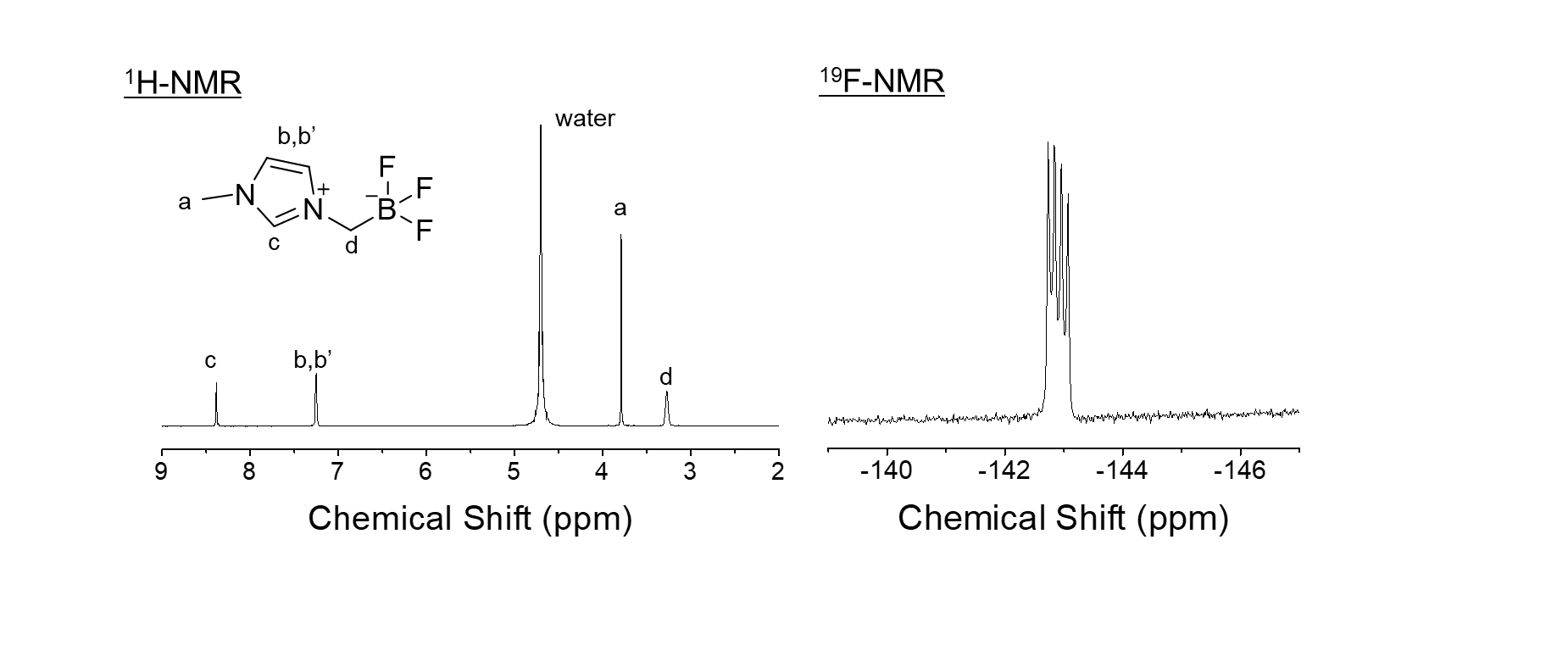
**

**Figure S2.** ^1^H-NMR and ^19^F-NMR spectra of ZImBF_3_.

((3-(1-methylpyrrolidin-1-ium-1-yl)propyl)sulfonyl)((trifluoromethyl)sulfonyl)imide (ZPyTFSI) and ((3-(3-methylimidazolium-1-yl) propyl)sulfonyl)((trifluoromethyl)sulfonyl) imide (ZImTFSI): Acetone was added to a mixture of trifluoromethanesulfonamide (5 g, 33.5 mmol) and distilled water (12 mL). Potassium carbonate (2.8 g, 20.3 mmol) was added and the mixture was stirred for 24 h. The resultant mixture was dissolved in acetone (80 mL) at 50 °C, the undissolved salt was removed by filtration. The filtrate was concentrated to give potassium trifluoromethylsulfonyl amide (KTFSA) as a white solid. The KTFSA (0.34 g, 1.8 mmol) was dissolved in acetonitrile (30 mL) and 3-chloro-1-propanesulfonyl chloride (0.34 g, 1.92 mmol) was added dropwise at 55 °C. Potassium carbonate (0.6 g, 4.3 mmol) was added and stirred for 30 h. Subsequently, the undissolved salt was removed by filtration and the filtrate was concentrated, washed with THF, and dried in vacuum, yielding potassium (3-chloropropylsulfonyltrifluoromethane sulfonyl)imide (KTFSI−Cl).^[3]^

For the synthesis of ZPyTFSI, KTFSI−Cl (0.5 g, 1.6 mmol) was dissolved in n-methylpyrrolidine (20 mL) and refluxed at 110 °C for 24 h. The mixture was purified by repeated recrystallization in methanol and then dried under vacuum to yield ZPyTFSI (0.29 g 54%). ^1^H NMR (500 MHz, D_2_O) δ_H_ (ppm): 3.46 (m, 4H, C**H**_2_NC**H**_2_), 3.28 (t, 2H, CH_2_C**H**_2_S), 3.00 (s, 3H, NC**H**_3_), 2.28, (m, 2H, CH_2_C**H**_2_CH_2_S) 2.14 (br, 4H, C**H**_2_CH_2_NCH_2_C**H**_2_), ^19^F NMR (470 MHz, D_2_O) δ_F_ (ppm): −77.68

**
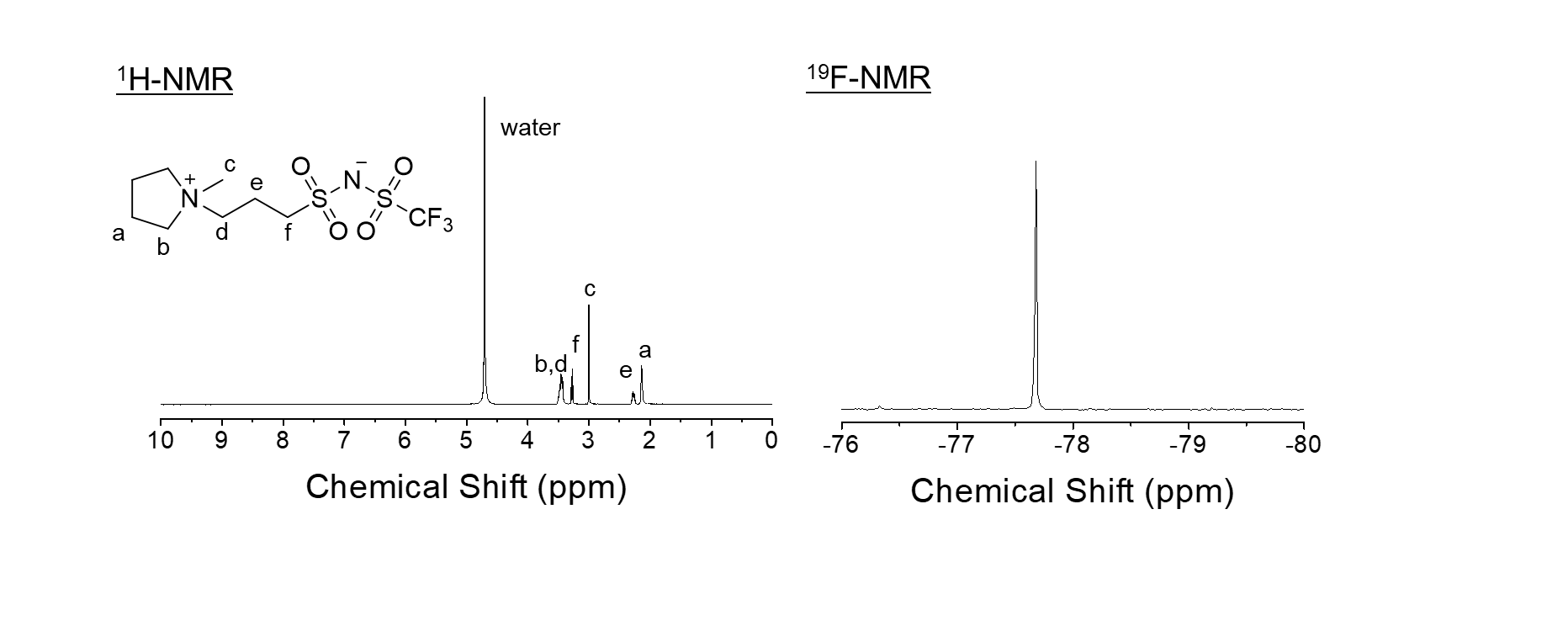
**

**Figure S3.** ^1^H-NMR and ^19^F-NMR spectra of ZPyTFSI.

For the synthesis of ZImTFSI, KTFSI−Cl (0.5 g, 1.6 mmol) was dissolved in 1-methylimidazole (25 mL) and refluxed at 120 °C for 48 h. The mixture was purified by recrystallization in methanol and distilled water. The crystallization was repeated three times and then ZImTFSI (0.25 g 47%) was finally obtained by drying under vacuum. ^1^H NMR (500 MHz, D_2_O) δ_H_ (ppm): 9.11 (s, 1H, N(C**H**)N), 7.78, 7.70, N(C**H**C**H**)N) 4.30 (t, 2H, NC**H**_2_CH_2_) 3.84 (s, 3H, N(C**H**_3_)), 3.01 (t, 2H, CH_2_C**H**_2_S), 2.21 (m, 2H, CH_2_C**H**_2_CH_2_S), ^19^F NMR (470 MHz, D_2_O) δ_F_ (ppm): −76.51

**
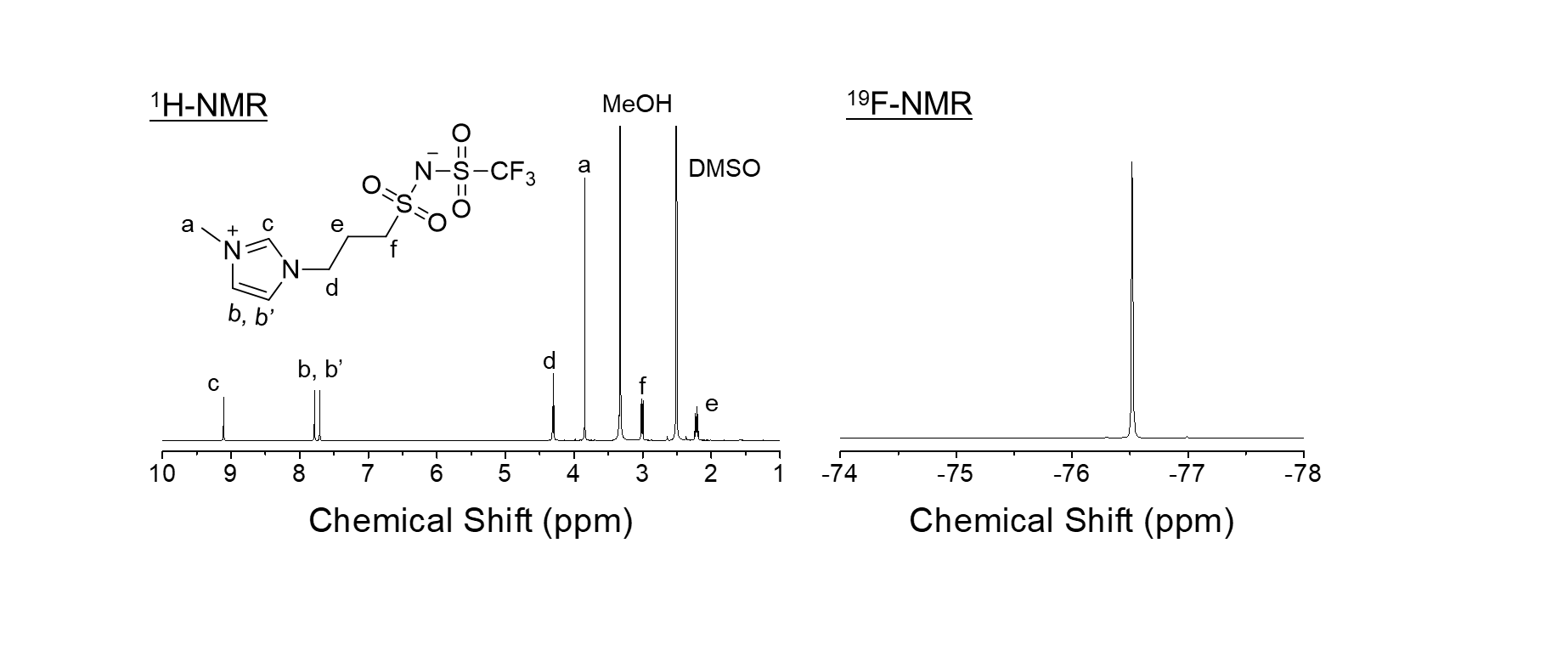
**

**Figure S4.** ^1^H-NMR and ^19^F-NMR spectra of ZImTFSI.

**X-ray scattering experiments**

All sample preparations were carried out inside an argon-filled glovebox to avoid water contamination of hygroscopic components. Pre-determined amounts of block copolymers and zwitterions were dissolved in THF (anhydrous, 99.9%)/methanol (anhydrous, 99.8%) mixtures (90/10 vol%) at a concentration of 5 wt%. The resulting mixtures were cast onto glass plates at 60 °C and subsequently dried under vacuum at 30 °C for one week. The delaminated films were positioned between two mirror-polished stainless steel electrodes with a 280 μm-thick Kapton spacer and hot-pressed to form membranes approximately 250 μm thick. The samples were then sealed in airtight aluminum cells with two Kapton windows. Synchrotron small-angle X-ray scattering (SAXS) and wide-angle X-ray scattering (WAXS) experiments were conducted at the Pohang Accelerator Laboratory (PAL) on beamlines 9A, 3C, and 4C, utilizing two-dimensional detectors. The incident X-ray beam had a wavelength (λ) of 0.063 nm (Δλ/λ = 10^-4^). The sample-to-detector distances were 3.0 m for SAXS, covering a scattering wave vector (*q*) range of 0.01 to 2.0 nm^-1^, and 20 cm for WAXS, with *q* range of 5.0 to 30 nm^-1^. For the temperature-dependent SAXS measurements, scattering profiles were recorded after equilibrating the sample at each temperature for 30 min.

**Transmission** **electron microscopy (TEM)**

Pre-determined amounts of block copolymers and zwitterions were dissolved in a THF (anhydrous, 99.9%)/methanol (anhydrous, 99.8%) mixtures (90/10 vol%) at a concentration of 0.25 wt% and cast onto Formvar-coated TEM grids inside an argon-filled glovebox. After vacuum drying for a week, the grids were exposed to ruthenium tetroxide vapor for 1 h. Bright-field TEM micrographs and electron energy loss spectroscopy (EELS) mapping images were acquired using a JEM-2200 FS (JEOL Ltd.) equipped with Cs-corrector and energy selecting slit, operating at 200 kV. All images analyses were performed using the Digital Micrograph software (Gatan Inc.)

**Dielectric relaxation spectroscopy**

Samples were prepared using the same procedure as those for the X-ray scattering experiments and were sandwiched between two freshly polished brass electrodes (20 mm diameter for the bottom electrode, 10 mm for the top electrode) with Kapton spacers. Prior to measurements, the electrode-sandwiched samples were annealed at 130 °C for 1 h to ensure good contact with the electrodes. Dielectric permittivity spectra were obtained using a Solartron 1260A impedance analyzer connected to 1296 dielectric interface, applying a 0.1 V sinusoidal voltage over a frequency range of 10^-1^–10^6^ Hz.

**Rheological measurements and thermal analysis**

Membranes approximately 500 μm thick were produced via hot pressing, and their rheological properties were recorded using the Discovery TA DHR-2 rheometer with a 0.8 cm parallel plate geometry. Temperature sweep measurements were performed at a strain of 0.1%, a heating/cooling rate of 1 °C min^-1^, and a frequency of 0.5 rad s^-1^.

Differential scanning calorimetry (DSC) thermograms of the samples were recorded over the temperature range of −70 to 150 °C using a TA Instruments (model Q20) at a heating rate of 10 °C min^–1^ and a cooling rate of 1 °C min^–1^. Approximately 10 mg of the sample was loaded in a standard aluminum pan inside an argon-filled glovebox, with an empty pan serving as a reference. Data from the second heating scan were analyzed and presented.

**Ultraviolet–visible spectroscopy**

Thin films of neat STMB, STMB doped with ZPyBF₃-0.5, and STMB doped with ZPyBF₃-1.0 were prepared by spin-coating of the samples onto quartz substrates. UV–Vis absorption spectra were recorded in the wavelength range of 200–900 nm using a Shimadzu UV-2600 spectrophotometer.

**Nanoindentation test**

Young’s modulus, hardness, and stiffness of neat STMB, STMB doped with ZPyBF₃-0.5, and STMB doped with ZPyBF₃-1.0 were evaluated using nanoindenter (KLA iNano nanoindenter) equipped with a diamond Berkovich tip. All tests were conducted under ambient conditions. The experiment was performed in a continuous stiffness measurement mode where a small oscillating load was applied at room temperature. The frequency, constant indentation strain rate, and displacement amplitude of the superimposed oscillating force were set as 100 Hz, 0.05 s^-1^, and 2 nm, respectively. The sample thickness was approximately 170 μm and the indentation depth for analysis was limited to less than 10% of the whole film thickness to eliminate the influence of the substrate. The thermal drift was kept below 0.05 nm s^-1^ during the testing. The average values were obtained for depths of 400–1000 nm, where the elastic modulus and hardness were consistent according to Oliver and Pharr method. Measurements were repeated 9 times at random points for each sample to ensure statistical significance.

**Density functional theory (DFT) calculations**

The geometries of monomeric units functionalized with -SO₃H or -TFSI groups, as well as zwitterions, were optimized using DFT. Binding energies between monomeric units and zwitterions were determined by calculating the stabilization energies of paired complexes. All DFT calculations were conducted with the Gaussian 09 software, employing the B3LYP functional and the 6-31G** basis set.

**Supporting Figures**


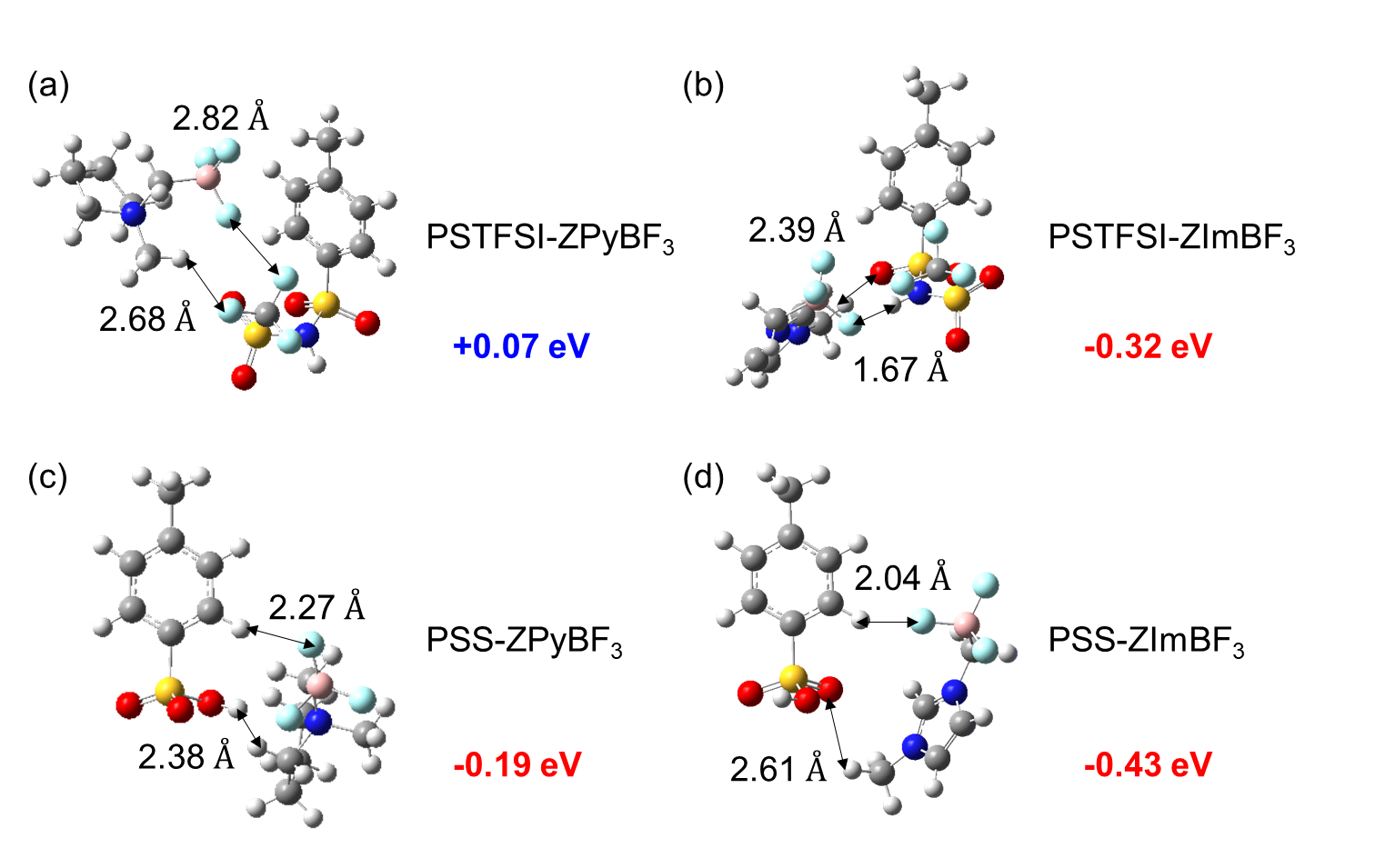


**Figure S5.** Predicted binding energies of monomeric units with zwitterions: (a) PSTFSI with ZPyBF_3_, (b) PSTFSI with ZImBF_3_, (c) PSS with ZPyBF_3_, and (d) PSS with ZImBF_3_.


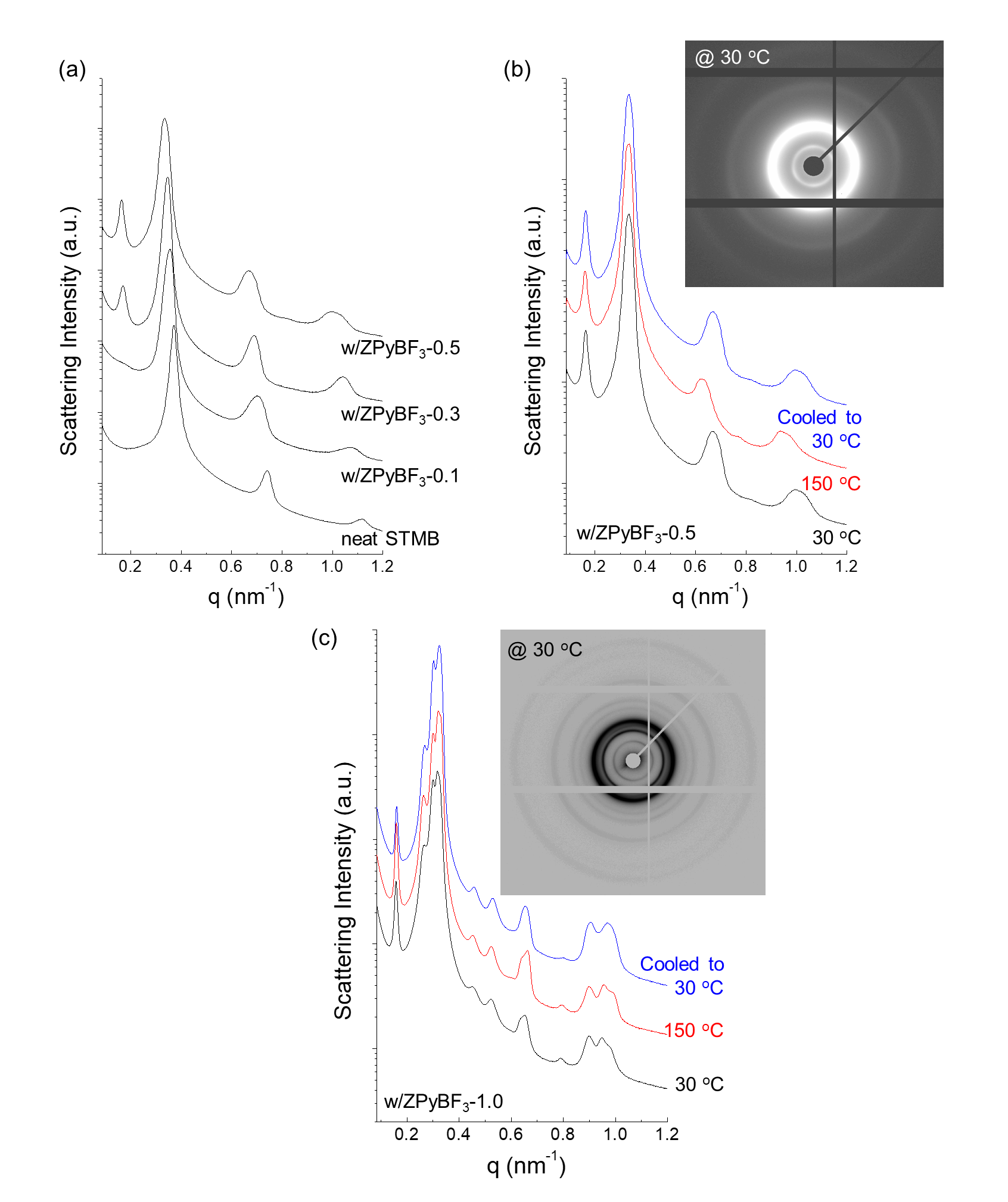


**Figure S6.** (a) SAXS profiles of STMB at different ZPyBF_3_ doping levels. (b, c) SAXS profiles of STMB doped with ZPyBF_3_-0.5 or ZPyBF_3_-1.0, measured with heating and after cooling back to 30 °C from 150 °C. 2D SAXS patterns of the cooled samples are shown in the insets.


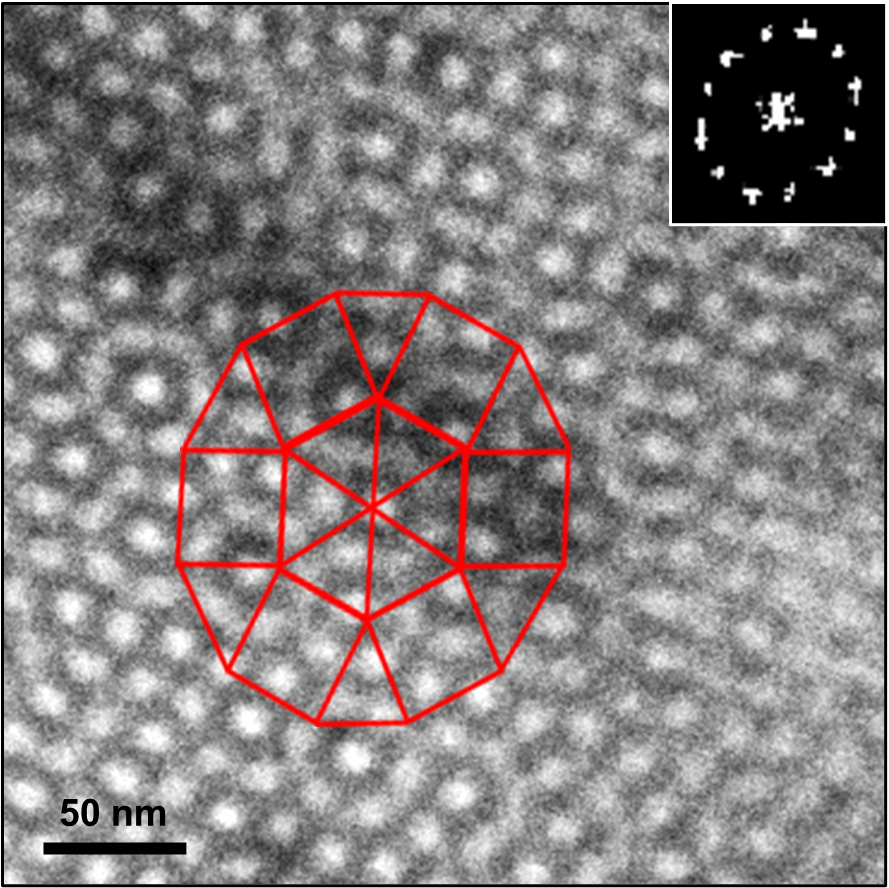


**Figure S7.** Bright-field TEM micrograph with corresponding FFT pattern for STMB with ZPyBF_3_-1.0, showing the DDQC phases.


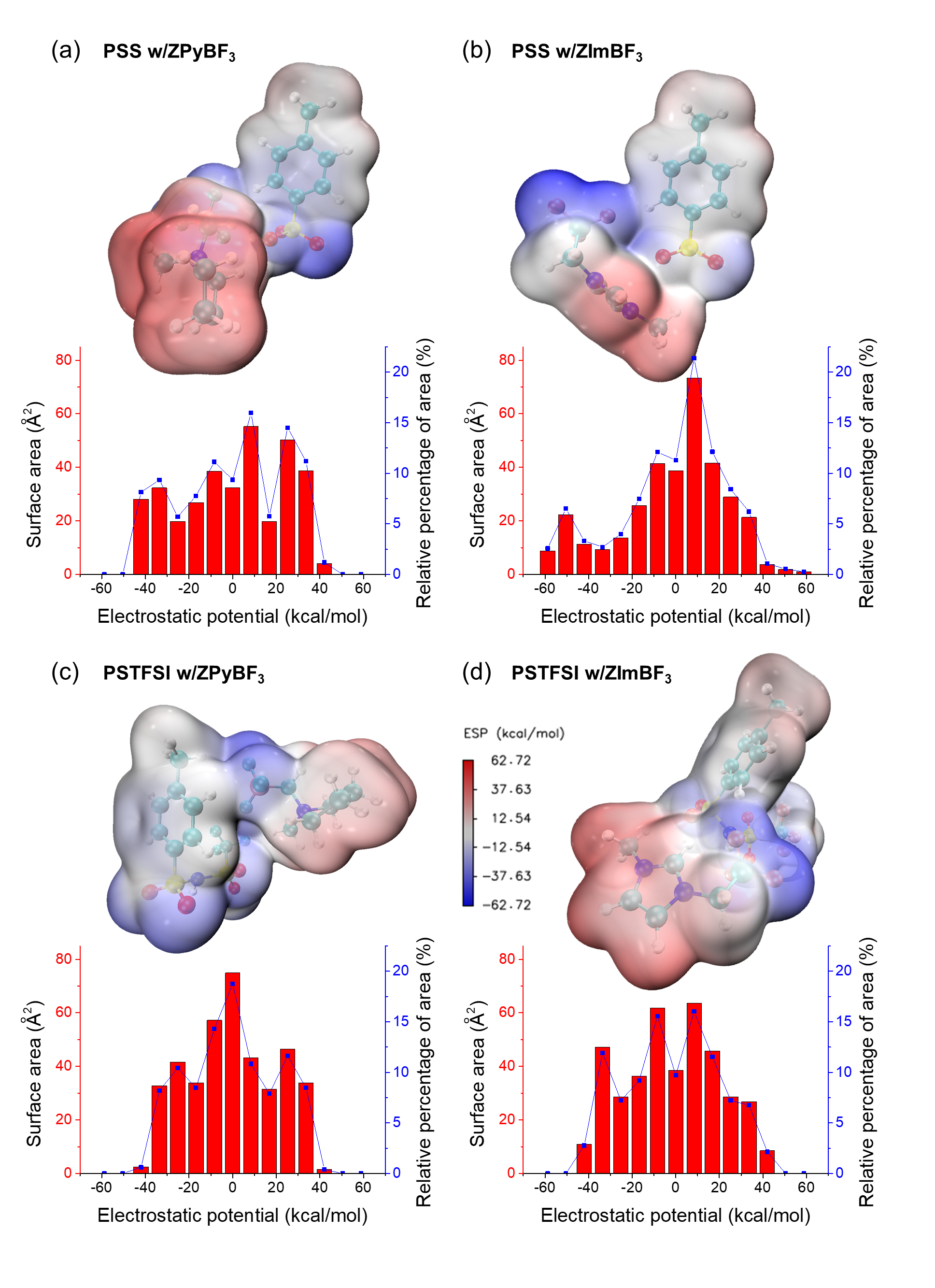


**Figure S8.** ESP analyses of monomeric units interacting with zwitterions: (a) PSS-ZPyBF_3_, (b) PSS-ZImBF_3_, (c) PSTFSI-ZPyBF_3_, and (d) PSTFSI-ZImBF_3_.

**
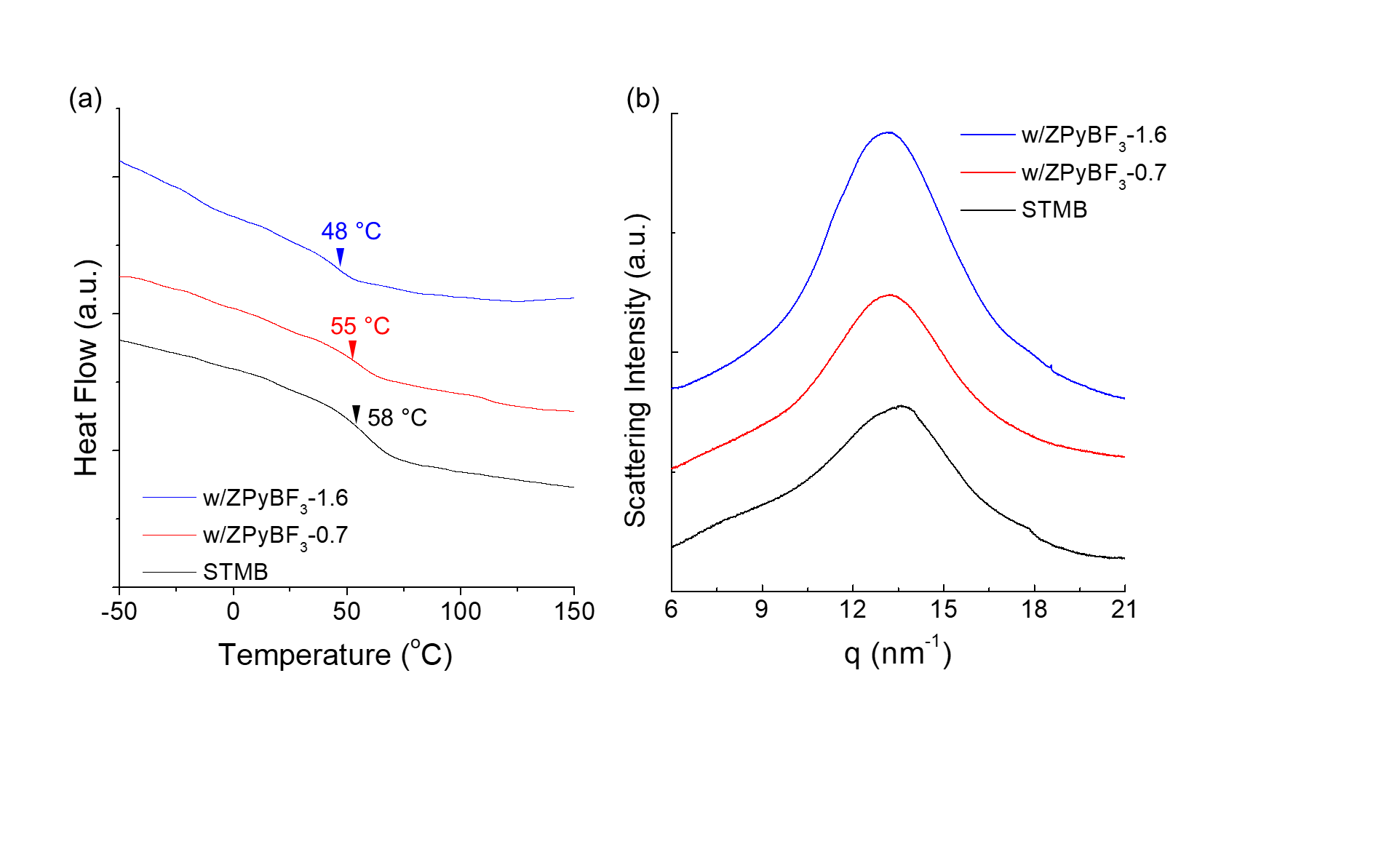
**

**Figure S9.** (a) DSC thermograms and (b) WAXS profiles of STMB doped with ZPyBF_3_.

**
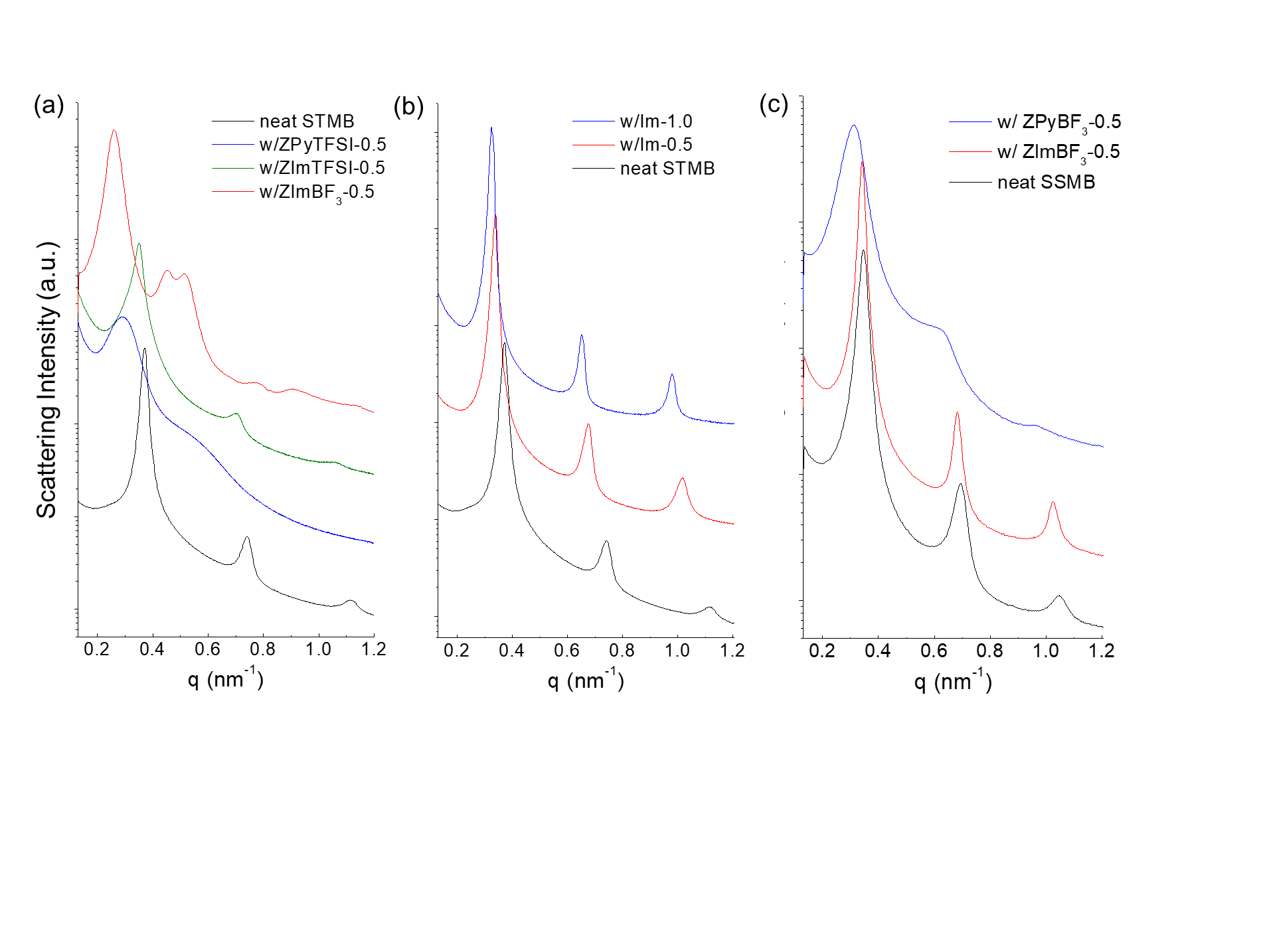
**

**Figure S10.** SAXS profiles of (a) zwitterion-doped STMB, (b) Im-doped STMB, and (c) zwitterion-doped SSMB, measured at 30 °C.

**
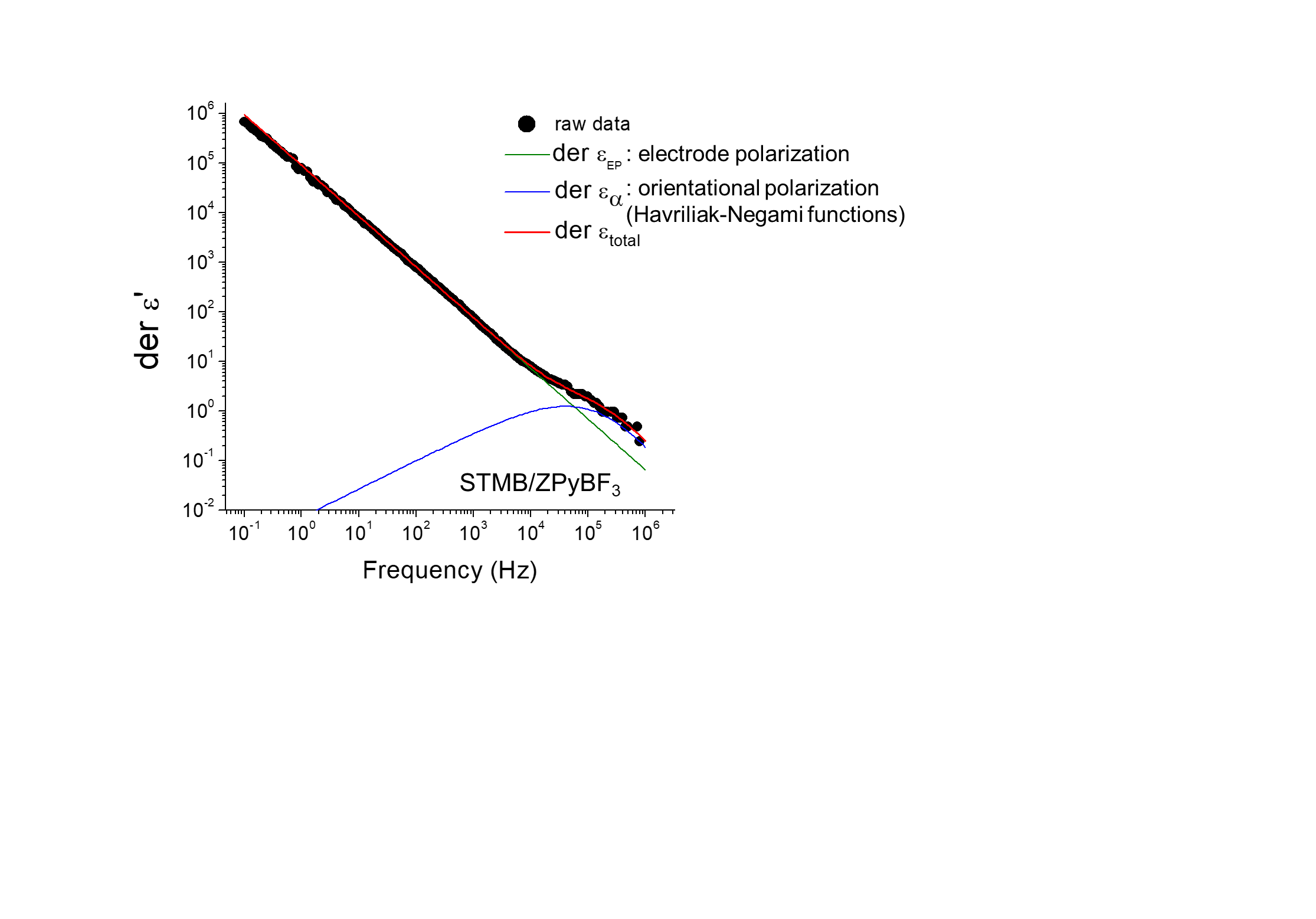
**

**Figure S11.** Dielectric permittivity spectra of STMB doped with ZPyBF_3_-0.5, measured at 25 °C, analyzed by accounting for electrode polarization and orientational polarization.

**
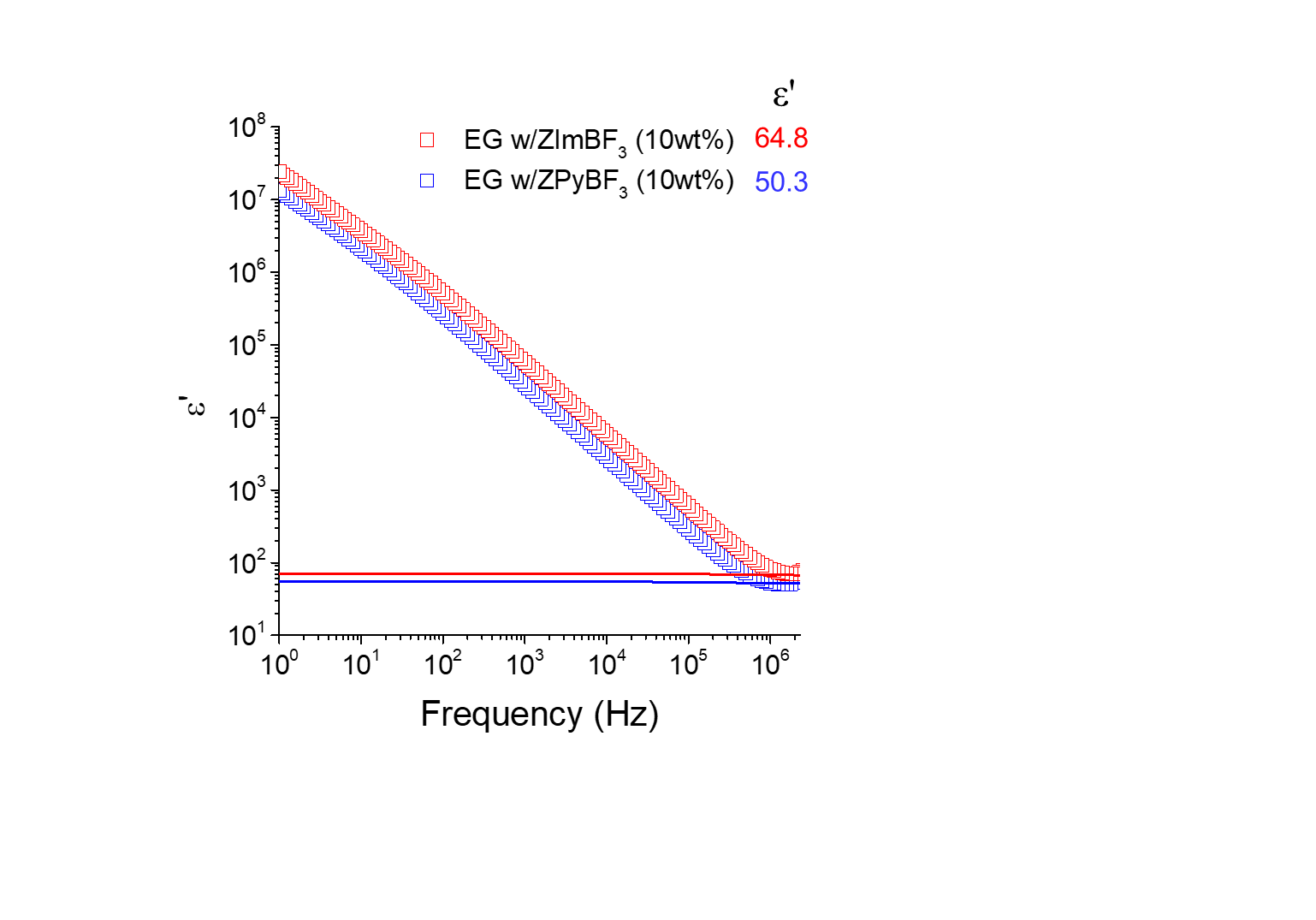
**

**Figure S12.** Dielectric permittivity spectra of ethylene glycol (EG) containing 10 wt% ZPyBF_3_ or 10 wt% ZImBF_3_, measured at 30 °C.

**References**

[1] M. J. Park, N. P. Balsara, *Macromolecules* **2008**, *41*, 3678-3687.

[2] F. Makhlooghiazad, L. A. O’Dell, L. Porcarelli, C. Forsyth, N. Quazi, M. Asadi, O. Hutt, D. Mecerreyes, M. Forsyth, J. M. Pringle, *Nat. Mater.* **2022**, *21*, 228-236.

[3] J. Foropoulos Jr., D. D. DesMarteau, *Inorg. Chem.* **1984**, *23*, 3720-3723.
